# In Silico Identification of Tripeptides as Lead Compounds for the Design of KOR Ligands

**DOI:** 10.3390/molecules26164767

**Published:** 2021-08-06

**Authors:** Azzurra Stefanucci, Valeria Iobbi, Alice Della Valle, Giuseppe Scioli, Stefano Pieretti, Paola Minosi, Sako Mirzaie, Ettore Novellino, Adriano Mollica

**Affiliations:** 1 Department of Pharmacy, University G. d’Annunzio Chieti, Via dei Vestini 31, 66100 Chieti, Italy; a.stefanucci@unich.it (A.S.); alice.dellavalle@unich.it (A.D.V.); giuseppe.scioli@unich.it (G.S.); 2 Department of Pharmacy (DIFAR), University of Genova, 16128 Genova, Italy; valeria.iobbi@edu.unige.it; 3 Centro Nazionale Ricerca e Valutazione Preclinica e Clinica dei Farmaci, Istituto Superiore di Sanità, Viale Regina Elena 299, 00161 Rome, Italy; stefano.pieretti@iss.it (S.P.); paola.minosi@uniroma1.it (P.M.); 4 Advanced Pharmaceutics and Drug Delivery Laboratory, Leslie L. Dan Faculty of Pharmacy, University of Toronto, 27 King’s College Circle, Toronto, ON M5S 1A1, Canada; sako.biochem@gmail.com; 5 NGN Healthcare, Via Nazionale Torrette, 207, 83013 Mercogliano, Italy; ettore.novellino@unina.it

**Keywords:** peptides, molecular modelling, k-opioid receptor, antinociceptive effect, binding

## Abstract

The kappa opioid receptor (KOR) represents an attractive target for the development of drugs as potential antidepressants, anxiolytics and analgesics. A robust computational approach may guarantee a reduction in costs in the initial stages of drug discovery, novelty and accurate results. In this work, a virtual screening workflow of a library consisting of ~6 million molecules was set up, with the aim to find potential lead compounds that could manifest activity on the KOR. This in silico study provides a significant contribution in the identification of compounds capable of interacting with a specific molecular target. The main computational techniques adopted in this experimental work include: (i) virtual screening; (ii) drug design and leads optimization; (iii) molecular dynamics. The best hits are tripeptides prepared via solution phase peptide synthesis. These were tested in vivo, revealing a good antinociceptive effect after subcutaneous administration. However, further work is due to delineate their full *pharmacological* profile, in order to verify the features predicted by the in silico outcomes.

## 1. Introduction

Opioids represent the most effective and widely used analgesics to treat acute and intense pain since ancient times. Most of them are selective agonists of G-coupled opioid receptors μ-, δ-, and k-opioid receptors (MOR, DOR and KOR respectively) [1]. Although opioid receptors are the best-known therapeutic targets for the treatment of acute and chronic diseases, their clinical use is limited by adverse side effects such as tolerance and dependence; thus, the development of analgesics with reduced side effects and lack of tolerance remains a main target in the field of medicinal chemistry [2]. ΚORs are considered an attractive target for the discovery of safe analgesics avoiding side effects including respiratory depression, tolerance, dependence, and constipation. They are widely expressed throughout the central and peripheral nervous system; among them dinorphins (encoded by the Pdyn gene) primarily activate the KORs with very low affinity for MOR and DOR [3]. In contrast to MOR and DOR receptor agonists, KOR agonists have been recognized as analgesics without addiction and tolerance insurgence, despite their tendency to induce dysphoria, anhedonia and hallucinations [4,5]. The crystal structure of human KOR in complex with the selective antagonist JDTic has been resolved in 2012 [6]. Che et al. provided the crystal structure of human KOR in complex with the agonist MP1104 in the active-state [7,8]. These clarify the conformational differences between inactive and active states, providing details on ligand–receptor interactions [7,8,9,10]. The activation of KOR by endogenous peptide or exogenous synthetic agonists is associated with behavioral and mood effects, including analgesia, sedation, and perceptual distortions [11,12,13], while antagonists binding at the same site block the activation of KOR; thus, they may be used for treatment of depression, anxiety, addictive disorders, and other psychiatric conditions [14,15].

KOR is the main subtype of opioid receptors responsible for mediating dynorphin-related actions and dynorphin-related peptides, such as stress, addiction, emotion, and perception. KOR agonists have also been shown to inhibit hyperalgesia induced by the μ agonist receptor [9,16]. Recent studies have uncovered further potential therapeutic areas for KOR ligands, such as affective disorders and addiction-related behaviors. As recently demonstrated for other GPCRs, structural insights from active and inactive receptors can be exploited in virtual ligand screening protocols providing new compounds as promising analgesics [17,18]. Several groups have shown that, unlike other opioid receptors, KOR agonists inhibit dopamine efflux in the mesolimbic system and block the gratifying effects of abuse drugs such as heroin and cocaine [19,20,21]. Most KOR agonists belong to five chemical classes: endogenous peptides (dynorphins), benzodiazepines (diazepam, tifluadom), benzazocines (bremazocine, pentazocine), arilacetamides (enadoline, U50488), and diterpenes (salvinorin A). Benzazocins, such as bremazocine, are not strictly selective KOR agonists, but they show strong analgesic effects. However, these molecules were discarded during clinical development due to psychotomimetic and dysphoric effects, although they had low tolerance potential and drug dependence [22,23]. KOR agonists were generally thought to exhibit adverse effects due to off-target action; thus, new k-selective agonists such as aryl-acetamide derivatives (enadolines, U69593, U50488) were developed to avoid psychotomimetic and dysphoric effects; however, they also produce hallucinations and aversion [24,25].

Salvinorin A, a very potent and selective KOR agonist is known for its psychedelic effects [26]. Despite such a different chemical structure, κ receptor agonists have more or less psychotomimetic effects, and, therefore, clinical development has failed. Not surprisingly, the simultaneous inhibition of multiple neurotransmitter systems by KOR agonists causes complex multidimensional effects, such as hallucination, dysphoria and analgesia [27].

In addition, agonists induce phosphorylation of protein kinase 3 (GRK3) receptors of the κ receptor in the *C*-terminal region and the subsequent recruitment of β-arrestins, which are scaffolding proteins leading to the phosphorylation of P38 MAPK [28,29]. The identification of G proteins not dependent from the activation of P38 MAPK in the serotoninergic neurons of the dorsal Rafe by the KOR agonist U50488 was a step forward into the elucidation of the mechanisms by which the receptor κ averages adverse effects. Interference on this signaling pathway in mice through receptor mutation (KORS369A), the deletion of GRK3 or the conditional cancellation of P38-MAPK blocks the adverse effects of κ agonists without reducing their analgesic effects [30,31,32,33]. These studies have important therapeutic implications because a selective partial κ receptor agonist that does not efficiently activate arrestin-dependent reporting could produce analgesia without significant dysphoria. In addition, the κ-receptor-mediated activation of the P38 MAPK in glia seems to be important for the development of hyperalgesia following peripheral neuropathy [34,35,36].

Roth and colleagues performed systematic pharmacological studies to discover a partial G receptor agonist, RB-64 (22-thiocyanatosalvinorin A) [26]. This study, using KO wild type and β-arrestin-2 mice, showed that RB-64-mediated G protein signaling induces analgesia and aversion, while β-arrestin-2 signaling averages sedation, anhedonia, and motor incoordination. This is a clear example of how the characterization of signaling pathways, which mediate specific behaviors, can ultimately be used for drug development. So far, almost all studies to determine the biased agonism of GPCR have investigated the partiality of a ligand for both protein G and β-arrestin, dependent only on downstream signaling.

**Figure 1:**
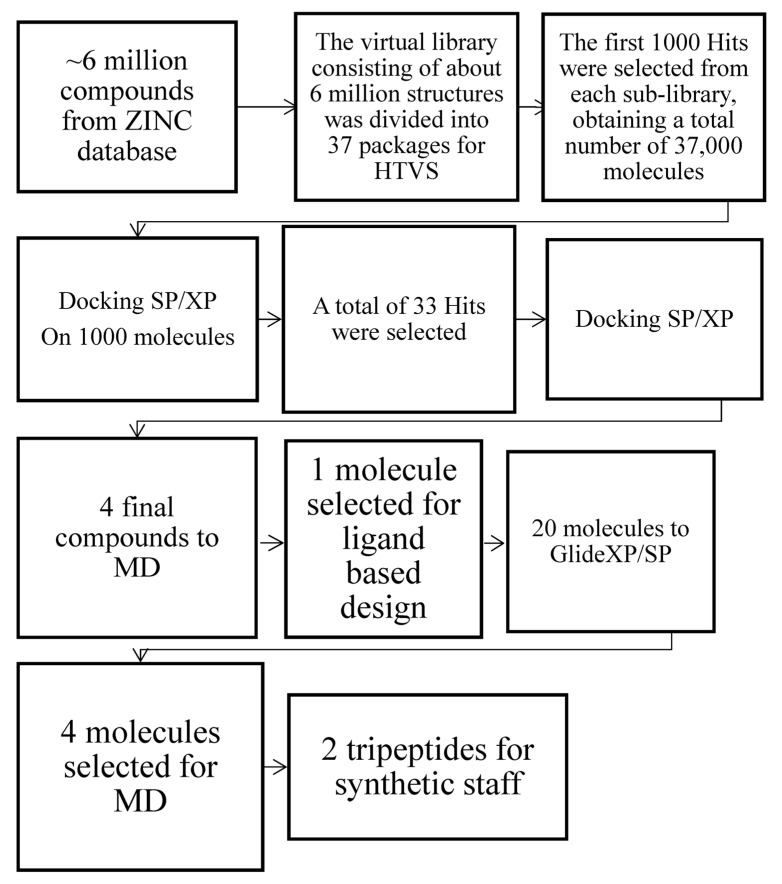
Computational workflow for the identification of the best two hits via Glide XP/SP and MD simulations.

Thus, diverse signals (e.g., phosphorylation of the PKC or GRK-dependent receptor) are a prerequisite. Over the years, several opioid ligands have been identified with the deconvolution of mixture-based combinatorial libraries at the Torrey Pines Institute of Molecular Studies (TPIMS) [37,38]. Computational studies through molecular scaffolds, molecular properties, and structural fingerprints show the diversity of these libraries and their uniqueness, based on: (a) the partial overlap with the structural space of drugs; (b) the presence of scaffolds not contained in other collections of compounds; (c) the increased molecular complexity compared to libraries of compounds commonly used in high-throughput screening (HTS) programs [39]. Structure-based drug design employs methods of receptor-based virtual screening (VS) and molecular docking for binding pose prediction, hit identification, and lead optimization. As part of our ongoing effort to discover new κ modulators with novel structures [40], the study herein is focused on the crystal structure of the KOR active-state for the discovery of novel KOR ligands using VS (Figure 1).

Our computational protocol allowed us to identify two best hits as tripeptides that were synthesized in solution following standard peptide protocol synthesis [41,42]. The two compounds obtained in modest yields and excellent purity were also tested in vivo.

A comparison with other opioid receptor structures identifies residues critical for KOR activation and highlights the key molecular characteristics of subtype selectivity and signal bias.

The basic scaffolds JDtic and MP1104 take distinctive poses, although with common characteristics typical of opioid ligands: (1) anchoring in the receptor binding pocket through a saline bridge with D138 in TM3; (2) interaction with TM5 through a phenolic group; (3) forming interactions with TM2/3 through chemically different portions [43,44]. The JDtic antagonist and the MP1104 agonist both form a saline bridge between their respective amino and D138 receptor patterns as observed in many GPCR-ligand complexes. The greater distance of this saline bridge (3.0 Å) compared to similar interactions in KOR-JDtic (2.6 Å) and MOR-BU-72-Nb39 (2.7 Å) involves a weaker ionic interaction between MP1104 and KOR. D138 also forms a hydrogen bonding network with T1112.56 and Y3207.43 in KOR-MP1104-Nb, which is probably critical for full KOR activation; additionally, the mutation of these residues strongly attenuate or delete β arrestin2-recruitment mediated by MP1104 or Dynorphin A 1–17, respectively. The phenolic groups MP1104 and JDtic extend towards TM5, forming hydrogen bonds mediated by water with the backbone of the K227 carbonyl oxygen. This interaction was proposed to simulate the *N*-terminal tyrosine found in endogenous opioid peptides [45,46,47].

Directing the orientation of a rigid and hindered structure inside the binding pocket is fundamental to determine the effectiveness/strength of the ligand by minor changes in contact forces or tensions generated by substituents [48]. The orientation within the pocket probably depends (i) on the hybridization of the intramolecular bonds that determine the angles between the functional modules of the compound and (ii) specific interactions of the receptor subtype. Consequently, even small changes to identical scaffolds can subtly affect the compound binding pose, its potency, and/or effectiveness, as observed for other GPCR ligands [49].

## 2. Results and Discussion

### 2.1. Structure Based Design

The dipeptide H-D-Tyr-Val-NH_2_ (ZINC71788314) obtained from virtual screening, presents interesting features: (a) a favorable docking score, with a value of −8.592; (b) structural simplicity, which allows an easy in silico optimization process and a feasible synthetic process; (c) amino-terminal tyrosine residue, essential for an optimal interaction with the opioid receptor [50]. Thus it was considered as the lead compound for the further development of KOR ligands. In the first attempt, a lipophilic portion was inserted, represented by a benzyl group bonded to the carboxy-terminal, in order to stabilize the ligand at the orthosteric site of the receptor through the formation of hydrophobic interactions [51]. The different combinations of the D and L amino acids in the dipeptide tyrosyl-valine benzyl ester were considered for the calculations with Glide SP and XP docking methods. Then the elongation of the dipeptide’s carboxy-terminal was taken in consideration, through the insertion of valine (Table 1) or tryptophan (Table 2) in the third position. The latter were considered by virtue of their chemical-physical properties, with the aim of creating a hydrophobic cluster [52]. The amino acid sequences of the two tripeptides, Tyr-Val-Val-OBz and Tyr-Val-Trp-OBz, were modified by D and L amino acids, and a docking score was calculated for each of them. The best result in terms of docking score values was obtained for the sequence D-Tyr-L-Val-L-Val-OBz, which was assumed as lead compound for further modification. The third approach consisted in the insertion of a bromine in *meta* position on the *C*-terminal aromatic ring. This modification was carried out with the aim of increasing the lipophilicity of the molecule intensifying the hydrophobic interactions between the *C*-terminal portion and the receptor pocket [53]. Both D and L series were considered in the docking prediction (Table 3).

**Figure 2:**
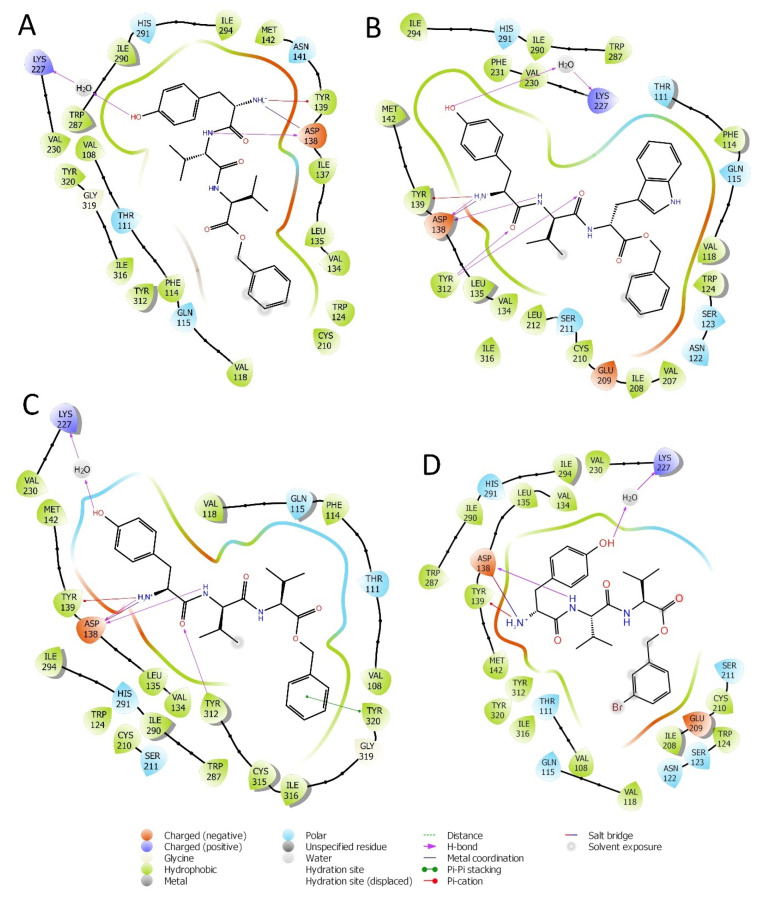
Interactions of peptides H-D-Tyr-Val-Val-OBz (**A**), H-D-Tyr-Val-Trp-OBz (**B**), H-D-Tyr-D-Val-Val-OBz (**C**), and H-D-Tyr-Val-Val-O-(3-Br)-Bz (**D**) with the amino acids residues of KOR binding site.

The peptides with the best docking score values were selected for the next phase of molecular dynamics (MD), which allows one to simulate and analyze the physical movements of atoms and groups of atoms within a molecular system. The final poses of the best tripeptides obtained by the Glide/XP docking method are reported below (Figure 2).

### 2.2. Molecular Dynamics Simulation

**Figure 3:**
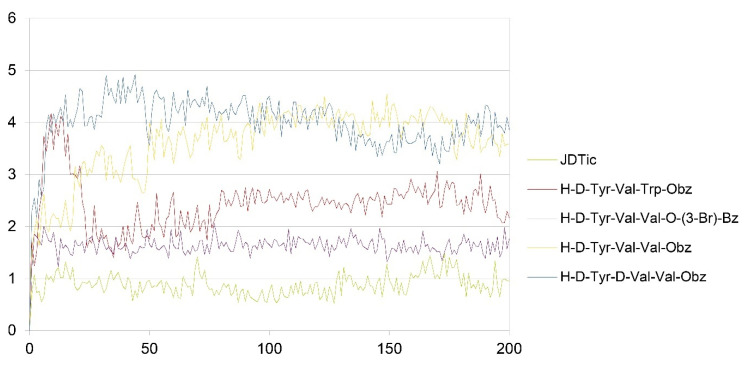
RMSD values of JDTic and the designed peptides (x axis: RMSD in Angstrom; y axis: time in ns).

**Figure 4:**
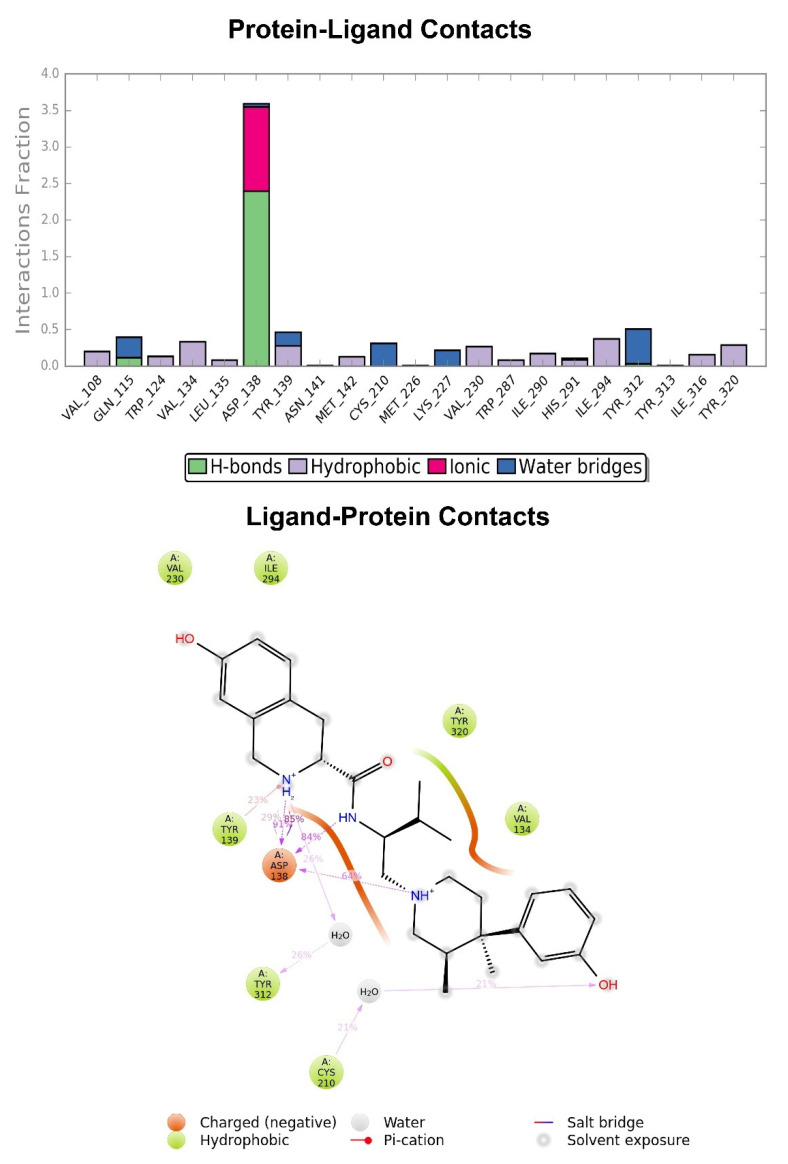
Graphic representation of the interactions between the JDTic and KOR binding site, expressed in %. Hydrogen bonds are in violet lines.

The simulation was conducted on the four peptides selected in the design phase: H-D-Tyr-Val-Val-OBz, H-D-Tyr-Val-Trp-OBz, H-D-Tyr-D-Val-Val-OBz, and H-D-Tyr-Val-Val-O-(3-Br)-Bz, which were submitted to the Desmond Molecular Dynamic System [54] feature and incorporated into Maestro 2017. RMSD analysis provides information on the stability of the ligand within the active site of the receptor (Figure 3 and Figure 4). The P-RMSF allows one to visualize the areas of the protein chain that fluctuate the most during the simulation, while the L-RMSF shows how the ligand fragments interact with the protein and determine its entropic role during the binding process. The bonds established between receptor and ligand have been evaluated and classified into four categories: (a) hydrogen bonds, (b) hydrophobic interactions, (c) ionic bonds, and (d) aqueous bridges, which mediate the interactions between the ligand and amino acid residues of the receptor. Below are the RMSD values of the four designed tripeptides and the crystallographic ligand (Figure 3).

**Figure 5:**
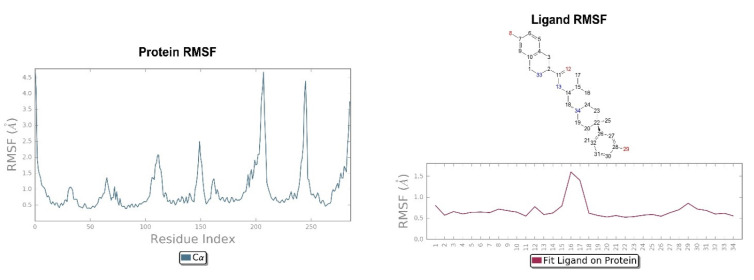
On the left: P-RMSF for KOR; on the right: L-RMSF of JDTic.

The crystallographic ligand has a stable pose inside the receptor pocket, as can be seen from the RMSD in Figure 3. The protein–ligand interactions are mainly represented by hydrogen bonds and the ionic nature with the residue of Asp138. The water bridge with the residue of Lys227, present both in the original pose and in the docked pose, was lost during the simulation (Figure 4). In the P-RMSF are reported the areas of the protein most affected by fluctuations, which exceed the value of 4.5 Å (Figure 5). JDTic does not show fluctuations greater than 1.0 Å, except for one of the two methyl groups linked to the isopropyl fragment (fragment 16), which reaches values greater than 1.5 Å (Figure 5).

**Figure 6:**
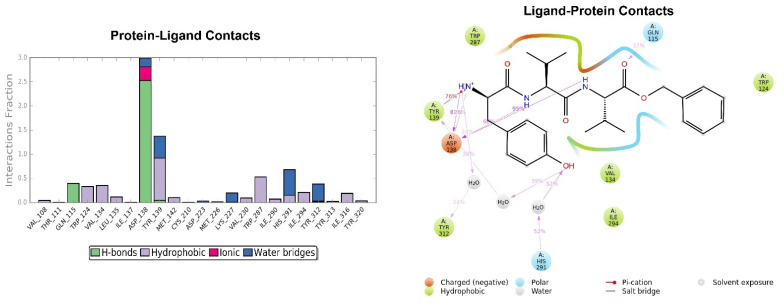
Representation of the main interactions found for H-D-Tyr-Val-Val-OBz in the KOR binding site, expressed in %. Hydrogen bonds are in violet lines.

**Figure 7:**
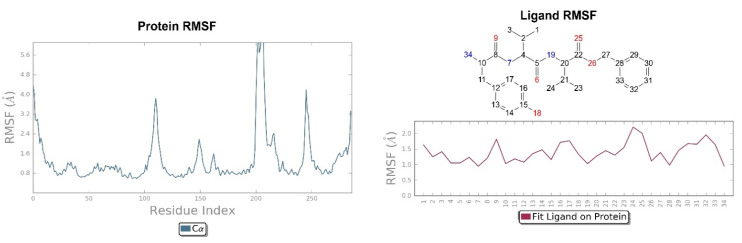
On the left: P-RMSF, receptor kappa; on the right: L-RMSF di H-D-Tyr-Val-Val-OBz.

The RMSD of the H-D-Tyr-Val-Val-OBz tripeptide located in the receptor active site appears to stabilize after 4 ns (Figure 3). The interactions with the KOR are better than in JDTic. In addition to the numerous hydrogen bonds and ionic interactions involving the Asp138 residue, there are further stabilizations of the ligand through different hydrophobic interactions with Tyr139 and Trp287 (Figure 6). Interestingly the water bridge is established between the phenolic hydroxyl group of D-Tyr and the Hys291 residue of the protein. The P-RMSF illustrates the fluctuations of the protein at slightly higher values (5.6 Å) than that found in JDTic (Figure 7). In the L-RMSF graph the best fluctuations are recorded for the side chain of the valine at the *C*-terminal end (fragment 24) (Figure 7).

**Figure 8:**
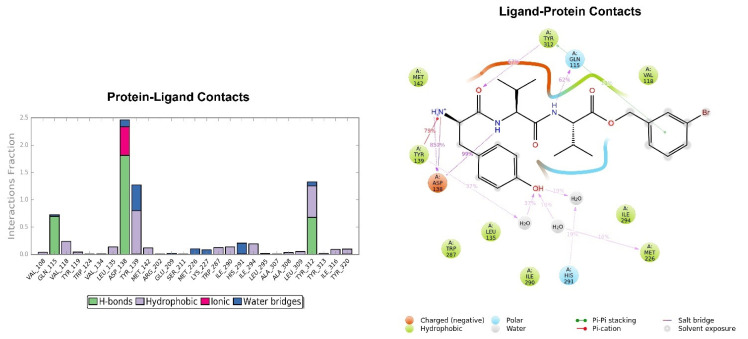
Key interactions of H-D-Tyr-Val-Val-O-(3-Br)-Bz (**6**) with KOR binding pocket expressed in %. Hydrogen bonds are in violet lines.

**Figure 9:**
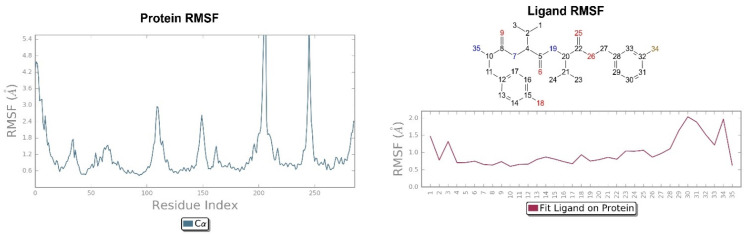
On the left: P-RMSF, KOR; on the right: L-RMSF of H-D-Tyr-Val-Val-O-(3-Br)-Bz (**6**).

Looking at Figure 8, the tripeptide H-D-Tyr-Val-Val-O-(3-Br)-Bz (**6**) turns out to be the ligand with the most stable profile during the simulation time. The receptor–ligand interactions are mainly characterized by hydrogen bonds with Asp138 and Gln115, with multiple hydrophobic interactions involving non-polar amino acid residues, such as Ile294 and Val118. Similarly to the tripeptide analyzed previously, there is interaction with the Hys291 residue assisted by a water molecule (Figure 8). The P-RMSF graph is comparable to the previous one (Figure 9); while the highest fluctuations are in correspondence with the aromatic ring replaced with the bromine atom (fragments 28–33 and 34).

**Figure 10:**
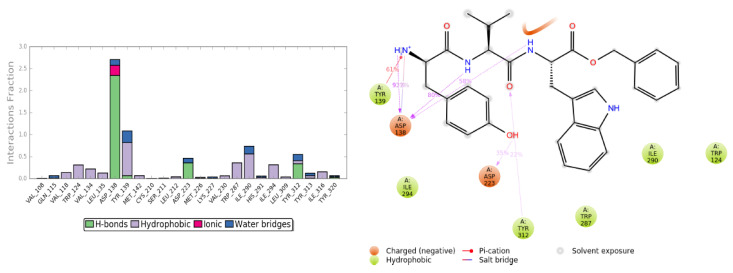
Interactions of H-D-Tyr-Val-Trp-OBz (**11**) within the KOR binding pocket, expressed in %. Hydrogen bonds are in violet lines.

**Figure 11:**
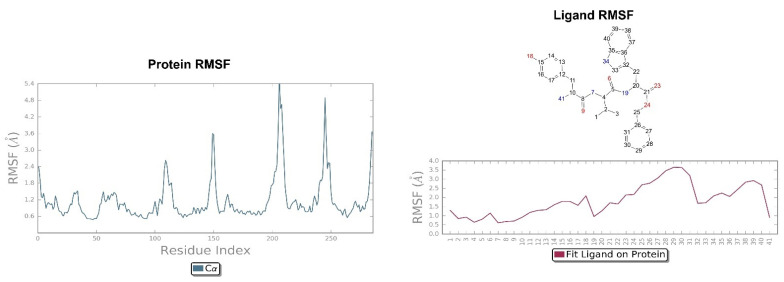
On the left: P-RMSF of KOR; on the right: L-RMSF of H-D-Tyr-Val-Trp-OBz (**11**).

The pose of H-D-Tyr-Val-Trp-OBz (**11**) is generally stable during molecular dynamics, and the binding with the KOR mainly focuses on hydrogen interactions with Asp138. The additional interactions of a hydrophobic nature allow for a good stabilization of the molecule within the receptor site; however, the hydrogen bond with the catalytic water molecule that acts as a bridge with the Lys227 residue is lost (Figure 10). The P-RMSF graph is comparable to the previous ones, and, in the L-RMSF, the main fluctuations are observed for fragments 25–31, due to the *C*-terminal benzyl group (Figure 11).

**Figure 12:**
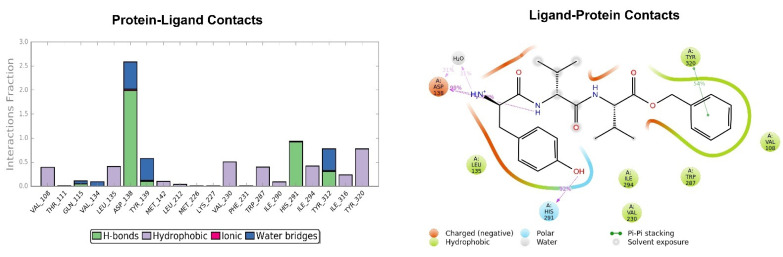
Interactions of H-D-Tyr-D-Val-Val-OBz within the KOR binding pocket, expressed in %. Hydrogen bonds are in violet lines.

**Figure 13:**
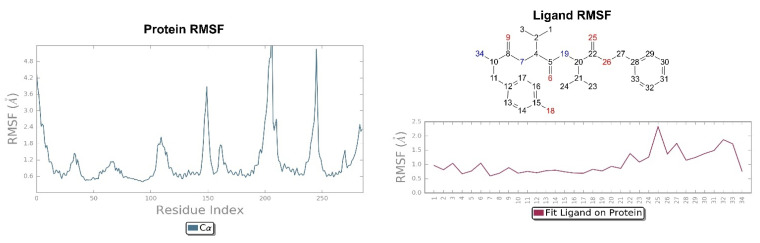
On the left: P-RMSF for KOR; on the right: L-RMSF of H-D-Tyr-D-Val-Val-OBz.

The pose of the tripeptide H-D-Tyr-D-Val-Val-OBz is stable and characterized by the prevalence of a hydrogen bond between the NH group of the backbone and the Asp138 residue. Interestingly the *N*-terminal group of tyrosine is involved in the hydrogen bond with Asp138 and a water molecule (Figure 12); the benzyl ring established a π-π stack interaction with Tyr320 and hydrophobic contacts with Val108, Trp287. The key interaction between the hydroxyl group of Tyr and His291 is also present. The highest fluctuations occur at the valine-O-benzyl portion (fragments 25–34) of the peptide (Figure 13).

To conclude tripeptides H-D-Tyr-Val-Val-O-(3-Br)-Bz (**6**) and H-D-Tyr-Val-Trp-OBz (**11**) are of great interest because they exhibit enhanced docking score values compared to the original dipeptide H-D-Tyr-Val-NH_2_ (−11.288 and −11.582 respectively, Table 2 and Table 3), higher than that of the crystallographic ligand (−11.176 with Glide/XP). The tripeptides designed *in silico* show strong stability, preserve the key interaction with the Asp138 residue, and are stabilized by efficient additional hydrophobic interactions. Thus, they were selected for solution phase peptide synthesis and further tested in vivo by means of tail flick and formalin tests.

### 2.3. Antinociceptive Effect In Vivo

**Figure 14:**
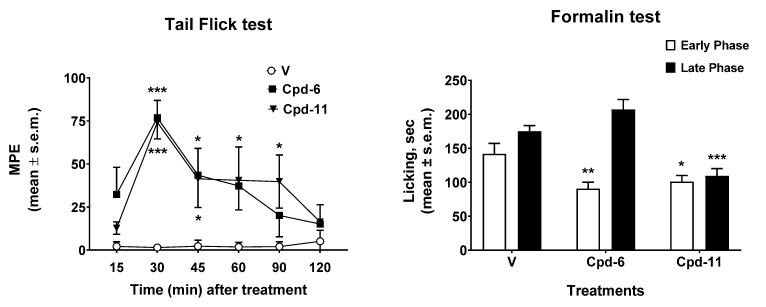
Effects induced by compounds **6** and **11** in the tail flick (left panel) and in the formalin (right test) tests. In the tail flick test, compounds were injected i.c.v. at a dose of 0.6 nmol/10 μL. In the formalin test, compounds were injected s.c. in the dorsal surface of the hind paw, 15 min before formalin. V is for vehicle-treated animals. * is for *p* < 0.05; ** is for *p* < 0.01; *** is for *p* < 0.001. *N* = 7.

The results obtained in the tail flick and in the formalin tests are reported in Figure 14. In the tail flick test, the administration of tripeptides **6** and **11** induced antinociceptive effects that peaked 30 min after the administration (Figure 14, left panel). After the peak time, compound **6** induced significant antinociceptive effect at 45 min after the administration, then its effect declined to MPE values similar to those observed in vehicle-treated animals 90–120 min thereafter. On the contrary, peptide **11** induced antinociceptive effects that were still significant at 45, 60, and 90 min after administration. The results obtained in the formalin test are reported in Figure 14, right panel. In the early phase of the formalin test, both **6** and **11** were able to reduce the nociceptive behavior induced by aldehyde. When the late phase was recorded, only compound **11** was able to reduce the licking nociceptive behavior induced by formalin, whereas compound **6** was ineffective. All together these data highlight a better antinociceptive effect for peptide **11** compared to **6** in both tests. The inflammation process contributes to the second phase of the test, during which compound **11** is still active, indicating an acute response to a model of ongoing acute pain involving inflammation and aspects of central sensitization. This could be due either to a good penetration of the blood–brain barrier ,as well as to the ability of this tripeptide to interact with opioid receptors at the periphery. With the aim of shedding some light on the possible pharmacokinetic profiles of these two compounds, an ADME study was performed in silico by means of SwissADME free web tool (http://www.swissadme.ch/index.php, accessed on 8 February 2021) [52].

### 2.4. In Silico ADME and Drug-Likeness Evaluation

The best two tripeptides **6** and **11** were submitted to an in silico evaluation of ADME (adsorption, distribution, metabolism, excretion) properties to estimate their pharmacokinetics and drug-likeness (Table 4).

Prediction of GIA is based on the brain or intestinal estimated permeation (BOILED-egg) model, which calculates the passive gastrointestinal absorption and blood–brain barrier penetration (Figures S5 and S6 in Supplementary Materials). This was high for peptide **6** and low for **11**; however, both of them show the same bioavailability score (0.55); this could be due to the fact that they turn out to be good substrates for P-glycoprotein, which is a cell membrane transport protein responsible for pumping drugs out [54,55,56]. Moreover, peptide **11** showed inhibition of CYP3A4, an enzyme involved in the metabolism of drugs [54], while peptide **6** lacks this interaction, which could prevent the accumulation of drugs. Peptides **6** and **11** have more than 10 rotatable bonds and a TPSA value > 130 Å (Table 4), indicating a low oral bioavailability [56]; in fact, both values of POLAR (TPSA) and FLEX for **6** and **11** are outside the desired range for improved bioavailability (Figures S5 and S6). Overall, this in silico study indicates slightly better pharmacokinetic properties for **6** compared to **11** but similar lipophilicity, which reflects their poor predicted bioavailability.

## 3. Methods and Materials

### 3.1. Library Preparation

The virtual library of compounds was retrieved from the ZINC database [57], from which a subset of ~6 million commercially available molecules were selected using the following filtering criteria: (i) molecules with molecular weight between 150 and 500; (ii) log P less than or equal to 5; (iii) hydrogen binding donor groups less than or equal to 5; (vi) hydrogen binding acceptor groups lower than or equal to 10; (v) PSA (molecular polar surface area) less than 150 and routable bonds less than or equal to 7. Compounds containing extremely reactive functional groups were discarded, e.g., thiol groups, Michael acceptors, and aldehyde groups.

The selected molecules in MOL2 format were submitted to LigPrep tool [58], incorporated in Maestro Schrödinger 2017-1 [59]. LigPrep allows one to generate accurate and energetically minimized 3D structures, following these parameters: (i) the addition of any absent hydrogen atoms; (ii) the removal of unwanted molecules (water, salts); (iii) the neutralization of all groups, before generating the states of ionization with the Epik [60] function, setting a pH range of 7.4 +/− 0.0, with the aim of replicating physiological conditions. The final steps of this process consist in the generation of a series of tautomers for each structure, preserving the stereochemistry of the chiral centers of the ligands.

### 3.2. Protein Preparation

The human KOR (PDB code 4DJH) was retrieved from PDB in complex with a selective antagonist JDTic ((3R)-7-hydroxy-*N*-[(2S)-1-[(3R,4R)-4-(3-hydroxyphenyl) -3,4-dimethylpiperidin-1-yl]-3-methylbutan-2-yl]-1,2,3,4-tetrahydroisoquinoline-3-carboxamide) [6]. The Protein Preparation Wizard tool embedded in Maestro 2017-1 [61] allows for the accurate conversion of the raw PDB file into a fully prepared protein structure. During this phase, hydrogen atoms were added to all protein residues, and three of the four chains were removed from the crystallized structure, retaining only the A chain containing the binding cavity. Then, hydrogen bonds and residue protonation states were refined by setting the pH to 7.4 with the PropKa function [62]. The minimization was carried out using the force field OPLS3 [63].

### 3.3. Receptor Grid Generation

The Receptor Grid Generation tool embedded in the Maestro 2017 suite was applied to form the grid that delimits the area of possible interactions between the ligand and the amino acid residues of the receptor. The kappa receptor and the crystallographic ligand (JDTic) are displayed in the Workspace, and an orthorhombic box is generated by exclusively centering the ligand, within which the virtual library of molecules will be anchored. A scaling factor of 1.00 on the Van der Waals radii of the non-polar atoms of the receptor was preserved, and the cutoff of the partial atomic charge was set at 0.25. A grid of 20 Å allows one to carry out the docking with ligands having dimensions comparable to the reference crystallographic ligand.

### 3.4. Glide Docking of the Co-Crystallized Ligand

**Figure 15:**
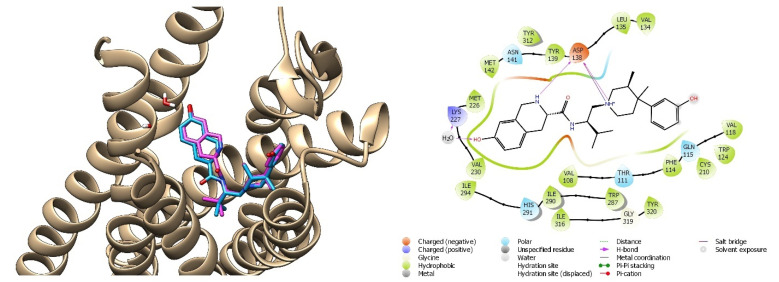
On the left: superimposition of the crystallographic ligand’s pose JDTic (pink) on the crystallized complex and its binding pose obtained with GLIDE/XP (blue); on the right: interactions of the HTVS binding pose of JDTic in the KOR binding cavity.

The crystallographic ligand was prepared with LigPrep, following the steps previously illustrated in paragraph 2.1. The JDTic antagonist was anchored to the active site of the kappa receptor through the Glide SP method, suitable for screening ligands with undefined quality, and subsequently with Glide XP [64]. The default parameters have been maintained and flexible docking has been opted for. The validation criterion of the docking method was the RMSD (root mean square deviation) value, i.e., the root of the mean square deviation, useful for calculating the average distance of structurally equivalent atoms. The calculated RMSD value, resulting from the overlap between the crystallographic ligand and the ligand prepared and repositioned in the active site by GlideXP, was found to be 0.119 Å. The JDTic crystallographic ligand was also subjected to HTVS with the aim to evaluate the interactions within the receptor pocket. Two key interactions were present, *e.g*., ionic interactions with the residue of Asp138 and the hydrogen bond between water molecule 1303 and Lys227 (Figure 15).

### 3.5. Virtual Screening Workflow

The virtual library consisting of about 6 million structures was divided into 37 packages or sub-libraries. The HTVS docking method was applied to each packet, designed to perform rapid screening operations on a large number of ligands. The first 1000 hits were selected from each sub-library, obtaining a total number of 37,000 molecules; among them, a first set was selected manually based on (i) the key interactions with the receptor: an ionic bond with Asp138 and a hydrogen bond with Lys227 assisted by a water molecule, (ii) docking score value, (iii) additional interactions with the kappa receptor: possible additional bonds, in addition to key interactions, were evaluated to favor a better pattern of interactions between the ligand and the active site of the receptor. (iv) RMSD value; (v) biological activity: the possible presence of biologically active molecular structures has been investigated in the literature. A total of 33 hits were selected, of which 10 with the best docking score values, 10 with interesting additional interactions, 10 with the best degree of overlap with JDTic and 3 with biological activity previously reported in literature [65,66,67]. Further docking optimization was done using Glide, which allows the ligand to be anchored to the active site of the receptor, providing for its binding mode. The previously created grid was selected, and two scoring functions with increasing precision were adopted: SP and XP Glidescore. We opted for a flexible docking model, leaving unchanged the standard Scaling factor parameters equal to 0.80 on the Van der Waals radii of the non-polar atoms of the receptor, defined as the atoms whose absolute value of the partial atomic charge (δ− o δ+) is positive and with a maximum value of 0.15. The ZINC04632302 outcome is a benzoimidazole that was characterized in vitro on MOR and DOR [68] and the compound ZINC06697859, which expressed antagonist activity with a high affinity for KOR (Ki = 0.09 μM) [69]. The ZINC71788314 is a D-tyrosyl-valinamide (H-D-Tyr-Val-NH_2_), a dipeptide obtained from the α-amidation process of the synthetic peptide D-tyrosyl-valyl-glycine (H-D-Tyr-Val-Gly-OH) in the brain [70].

### 3.6. Molecular Dynamics

The simulation was conducted on the four peptides H-D-Tyr-Val-Val-OBz, H-D-Tyr-Val-Trp-OBz, H-D-Tyr-D-Val-Val-OBz, and H-D-Tyr-Val-Val-O-(3-Br)-Bz through the Desmond Molecular Dynamic System [60,61,62,63,64,65,66,67,68,69,70,71] feature incorporated into Maestro 2017-1.

The system builder instrument in Desmond was used for the preparation of receptor–ligand complexes; the lipid bilayer membrane DPPC was set at 325 K, through which the various complexes to be examined were inserted. The entire system was centered by an orthorhombic box of 302,956 Å^3^ after minimization, which was saturated with water molecules by setting the TIP3P aqueous solvent model, in order to recreate physiological conditions. In the “Ions” section, the NaCl salt at a concentration of 0.15 M was added and the OPLS3 force field set.

The resulting system, displayed in the Workspace, was loaded in the “Molecular Dynamics” panel, belonging to the Desmond package. For each protein–ligand system, the overall simulation time was 20 ns, with an approximate number of frames equal to 200. For the ensemble option, which represents the macroscopic conditions of the system, the statistical set NPT (ensemble isothermal-isobaric), characterized by constant values of the number of particles, pressure, and temperature. In each molecular dynamics, the following parameters have been set: (a) temperature at 309.15 K and (b) pressure at 1.01325 bar. Newton’s equations of motion in the molecular dynamics trajectory were integrated with the r-RESPA method. Constraints have been placed on atoms capable of participating in hydrogen interactions, as they determine higher frequency vibrations, causing a restriction of the integration step (time-step) to about 0.5fs in an MD. Therefore the SHAKE method was used, reaching an integration step of 2 fs [70]. The contribution of the short range electrostatic interactions and the Lennard-Jones potential was evaluated by applying a spherical cut-off of 10 Å. Instead, the contribution of long-range electrostatic interactions, represented by the summation of the pairs of unbound atoms of the system, was estimated using the particle mesh Ewald method [71,72].

### 3.7. Chemistry

#### 3.7.1. General

All the reagents and amino acids for the synthesis were purchased from Sigma Aldrich (Milan, Italy). Final products were purified in RP-HPLC using a Waters XBridge^TM^ Prep BEH130 C18 column, 5.0 μm, 250 mm × 10 mm at a flow of 4 mL/min, and a Waters 600 binary pump (Milford, MA, USA), using as eluent a linear gradient of H_2_O/ACN 0.1% TFA, starting from 5% ACN to 90% ACN in 35 min. The nature of the protected N^α^-Boc(D)Tyr-Val-Val-O-(3-Br)-Bz and N^α^-Boc(D)Tyr-Val-Trp-OBz was confirmed by NMR with a Varian Inova 300 MHz instrument and ESI-LRMS Thermo Finnigan mass spectrometry (Somerset, NJ, USA). The purity of all final products as TFA salts was confirmed by NMR analysis, ESI-LRMS, and analytical RP-HPLC (C18-bonded 4.6 × 150 mm; 1 mL/min; 0.1% H_2_O/ACN gradient TFA from 5% to 95% ACN in 30 min), and the results were ≥95%.

#### 3.7.2. Synthesis

**Scheme 1:**
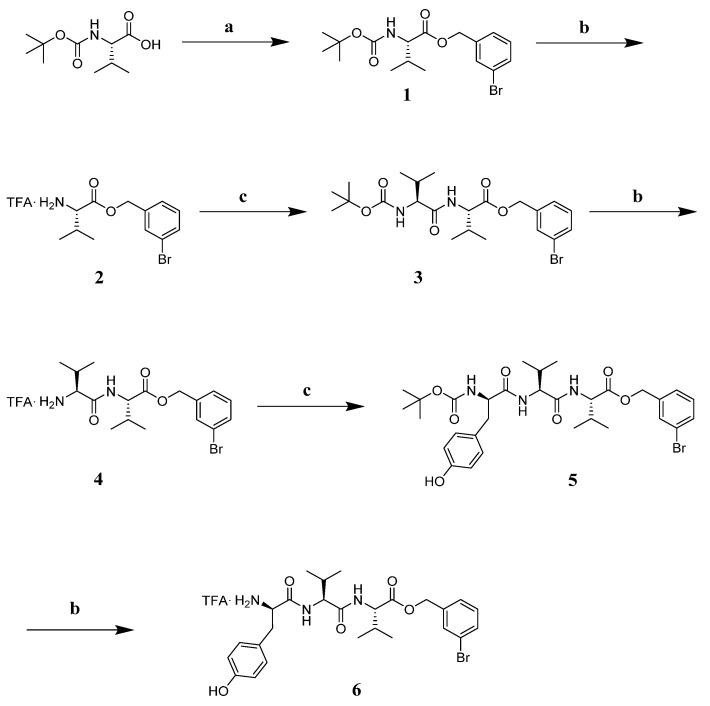
Reagents and conditions: (**a**) K_2_CO_3_ (1.2 eq), 3-bromo-benzyl (1 eq), ACN (5 mL), reflux, 4 h; (**b**) TFA:DCM = 1:1, r.t., 1 h; (**c**) EDC^.^HCl (1.1 eq), HOBt (1.1 eq), Boc-Val-OH for **2** or Boc-Tyr-OH for **5** (1 eq), DIPEA (3 eq), in DMF r.t. 12 h.

The tripeptide TFA^.^NH_2_-(D)Tyr-Val-Val-O-(3-Br)-Bz (**6**) was obtained starting from Boc-Val-OH, which was involved in a benzylation reaction with 3-bromo-benzyl, K_2_CO_3_ at reflux in ACN for 4 h. Intermediate **1** was deprotected with a mixture TFA:DCM = 1:1 at r.t. under stirring for 1 h. Intermediate **2** was reacted with Boc-Val-OH, EDC^.^HCl, HOBt and DIPEA in DMF at r.t. for 2 h. Repeated deprotection and coupling steps afforded Boc-protected intermediate **5** [73]. This peptide was treated with the TFA/DCM mixture so as to obtain the desired final product **6** as a TFA salt (Scheme 1).

**Scheme 2:**
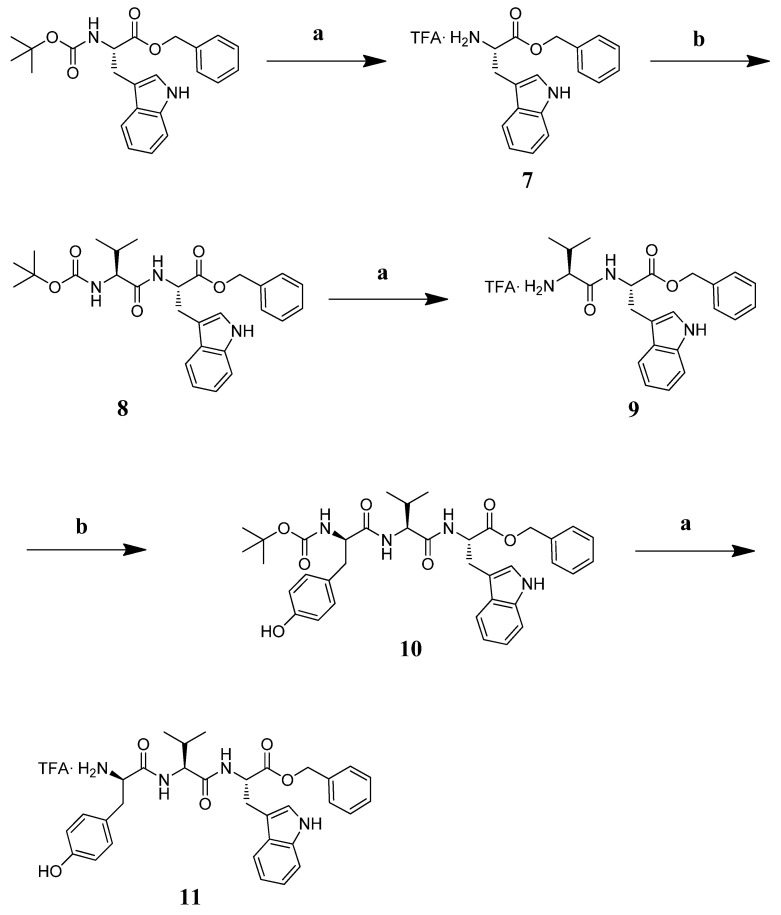
Reagents and conditions: (**a**) TFA:DCM = 1:1, r.t. 1 h; (**b**) EDC·HCl (1.1 eq), HOBt (1.1 eq), Boc-Val-OH or **9**, or Boc-D-Tyr-OH (1 eq) for **10**, DIPEA (3 eq), in DMF, r.t. 12 h.

The tripeptide TFA·NH_2_-D-Tyr-Val-Trp-OBz (**11)** was prepared in solution starting from Boc-Trp-OBz. The amino acid was deprotected with a mixture of TFA: DCM = 1: 1 at r.t. for 1 h and subsequently reacted with Boc-Val-OH in DMF, EDC^.^HCl, HOBt, and DIPEA at r.t. for 12 h. Intermediate **8** was deprotected and reacted with Boc-D-Tyr-OH under the aforementioned conditions. The final product **11** was obtained following the removal of the *tert*-butyloxycarbonyl group with an overall yield of 5.86% (Scheme 2).

#### 3.7.3. Characterization of Peptides and Intermediates

Boc-Val-O-(3-Br)-Bz (**1**). Boc-Val-OH (100 mg, 0.46 mmol) was dissolved in ACN (5 mL) with K_2_CO_3_ (69.65 mg, 1.2 eq) and 3-bromo-benzyl (104.97 mg, 1 eq). The mixture was left to reflux for 4 h., checking the completeness of the reaction with TLC (100% DCM). The solvent was removed in vacuum giving a raw material pure in TLC (100% DCM, R*f* = 0.3) in 92% yield. ^1^H NMR (CDCl_3_) δ: 7.45 (m, 2H, aromatics); 7.25–7.20 (m, 2H, aromatics); 5.17–4.98 (m, 3H, NH^α^ + CH_2_ benzyl), 4.27 (m, 1H, CH^α^), 2.13 (m, 1H, CH^β^), 1.43 (s, 9H, Boc), 0.94 (d, 3H, CH_3_), 0.84 (d, 3H, CH_3_).

TFA^.^NH_2_Val-O-(3-Br)-Bz (**2**). Intermediate **1** was treated with a mixture of TFA:DCM = 1:1 stirring for 1 h at r.t. then the solvent was removed in a rotary evaporator, washed with DCM (5 times), dried in high vacuum, and used as such in the next step without further purification.

Boc-Val-Val-O-(3-Br)-Bz (**3**). Boc-Val-OH (90.81 mg, 1.1 eq) was dissolved in DMF (15 mL) at 0 °C, stirring for 5 min, then EDC·HCl (80.51 mg, 1.1 eq) and HOBt (56.75 mg, 1.1 eq) were added. After 10 min, intermediate **2** (149.10 mg, 0.38 mmol) and DIPEA (0.2 mL, 3 eq) were added, stirring at 0 °C for 10 min., then at r.t. for 12 hs. After a standard work-up, the desired peptide was obtained in 68% yield, pure in TLC (DCM:AcOEt = 1:1; R*f* = 0.2).

TFA^.^NH_2_-Val-Val-O-(3-Br)-Bz (**4**). Intermediate **3** was deprotected following the same procedure reported for intermediate **2**, and used as is without further purification.

Boc-D-Tyr-Val-Val-O-(3-Br)-Bz (**5**). Dipeptide **4** as a TFA salt was coupled with Boc-D-Tyr-OH (84.39 mg, 1.1 eq), following the procedure previously reported for peptide **3**. The Boc-protected peptide was purified on silica gel column (mobile phase AcOEt:DCM = 1:1). The desired product was obtained with 39.7% yield. LRMS for C_31_H_42_BrN_3_O_7_: clcd *m*/*z*: 647.2; found: 670.1 [M + Na]^+^.

TFA·NH_2_-D-Tyr-Val-Val-O-(3-Br)-Bz (**6**): Peptide **5** was treated with a TFA/DCM mixture following the procedure previously described, so as to give final peptide **6** as a TFA salt, the identity of which was confirmed through ^1^H-NMR. ^1^H-NMR (CDCl_3_) δ: 7.45 (m, 2H, aromatics); 7.25–7.20 (m, 9H, aromatics + NH_3_^+^); 6.86 (d, 2H, aromatics); 6.61 (dd, 2H, 2*NH^α^); 5.17–4.98 (m, 3H, NH^α^ + CH_2_ benzyl), 4.57 (m, 1H, CH^α^ Val); 4.35 (m, 1H, CH^α^ Tyr); 4.27 (m, 1H, CH^α^ Val), 2.93 (m, 2H, CH_2_^β^ Tyr); 2.13 (m, 1H, CH^β^ Val), 2.02 (m, 1H, CH^β^ Val); 0.94 (d, 6H, CH_3_), 0.84 (d, 6H, CH_3_).

TFA·NH_2_-Trp-OBz (**7**). Boc-Trp-OBz was treated with a mixture of TFA:DCM = 1:1 at r.t. for 1 h and used as such for the next reaction.

Boc-Val-Trp-OBz (**8**). Boc-Val-OH (40.6 mg, 1.1 eq) was dissolved in DMF (7 mL) in an ice bath, stirring for 5 min. EDC·HCl (36.42 mg, 1.1 eq) and HOBt (25.68 mg, 1.1eq) were added; after 10 min, intermediate **7** (1.1 mg, 1 eq) and DIPEA (3 eq) were transferred into the reaction round bottom flask left at 0 °C for 10 min and at r.t. overnight. The desired product was obtained after a standard reaction work-up in 85% yield, pure in TLC (AcOEt:DCM = 1:9, R*f* = 0.3).

TFA·NH_2_-Val-Trp-OBz (**9**). The Boc-protecting group was removed from **8** following the same procedure adopted for **7**.

Boc-D-Tyr-Val-Trp-OBz (**10**). The coupling reaction among intermediate **9** (66 mg, 0.13 mmol) and Boc-Tyr-OH (39.38 mg, 1.1 eq) was setup following the procedure previously described. The so obtained crude peptide was purified on silica gel column (mobile phase 1:1 AcOEt:DCM) affording the pure peptide in 19.93% yield (AcOEt:DCM =1:1; R*f* = 0.3). The identity was confirmed by LRMS and ^1^H-NMR. LRMS for C_37_H_44_N_4_O_7_: calcd *m*/*z*: 656.3; found *m*/*z*: 679.2 [M + Na]^+^. ^1^H-NMR (CDCl_3_) δ: 8.27 (s, 1H, NH indolic); 7.51 (d, 1H, Trp indolic); 7.32–7.07 (m, 6H, aromatics Trp + NH^α^ Trp + NH^α^ Val); 6.68 (d, 2H, Tyr aromatics); 6.48 (d, 2H, Tyr aromatics); 6.32 (d, 1H, Boc NH); 4.92 (s, 1H, CH_2_ benzyl); 4.94–4.90 (m, 1H, CH^α^ Tyr); 4.19–4.17 (m, 2H, CH^α^ Trp, Val); 2.95–2.88 (m, 4H, CH^β^ Tyr, Trp); 1.96 (m, 1H, CH^β^ Val); 1.39 (s, 9H, 3*CH_3_); 0.79–0.65 (dd, 6H, 2*CH_3_ Val).

TFA.NH_2_-D-Tyr-Val-Trp-OBz (**11**). Boc-D-Tyr-Val-Trp-OBz **10** was treated with a mixture of TFA/DCM = 1:1 at r.t. for 1 h. The so-obtained product as a TFA salt was purified on RP-HPLC, and the structure was confirmed with ^1^H-NMR. ^1^H-NMR (CDCl_3_) δ: 10.96 (s, 1H, OH Tyr); 8.27 (s, 1H, NH indolic); 7.99 (s, 3H, NH_3_^+^ Tyr); 7.51 (d, 1H, Trp indolic); 7.32–7.07 (m, 6H, aromatics Trp + NH^α^ Trp + NH^α^ Val); 6.68 (d, 2H, Tyr aromatics); 6.48 (d, 2H, Tyr aromatics); 4.92 (q, 1H, CH_2_ benzyl); 4.94–4.90 (m, 1H, CH^α^ Tyr); 4.19–4.17 (m, 2H, CH^α^ Trp, Val); 2.95-.88(m,4H, H^β^ Tyr, Trp); 1.96 (m, 1H, CH^β^ Val); 0.79–0.65 (dd, 6H, 2*CH_3_ Val).

### 3.8. In Vivo Assays

#### 3.8.1. Animals

In our experiments, we used CD-1 male mice (Charles River, Italy, Sant’Angelo Lodigiano, 25–30 g) maintained in colony, housed in cages (7 mice per cage) under standard light/dark cycle (from 7:00 a.m. to 7:00 p.m.), temperature (21 ± 1 °C) and relative humidity (60% ± 10%) for at least 1 week. Food and water were available *ad libitum*. The Service for Biotechnology and Animal Welfare of the Istituto Superiore di Sanità and the Italian Ministry of Health authorized the experimental protocol according to Legislative Decree 26/14.

#### 3.8.2. Treatment Procedure

DMSO was purchased from Merck (Rome, Italy). Peptide solutions were freshly prepared using saline containing 0.9% NaCl and DMSO in the ratio DMSO/saline 1:5 *v/v* every experimental day. These solutions were injected at a volume of 10 μL/mouse for intracerebroventricular (i.c.v.) administrations or at a volume of 20 μL/mouse for subcutaneous (s.c.) administrations.

#### 3.8.3. Surgery for Intracerebroventricular Injection

For i.c.v. injections, mice were lightly anesthetized with isoflurane, and an incision was made in the scalp, and the bregma was located. Injections were performed using a 10 μL Hamilton microsyringe equipped with a 26-gauge needle, 2 mm caudal and 2 mm lateral from the bregma at a depth of 3 mm.

#### 3.8.4. Tail Flick Test

The tail flick latency was obtained using a commercial unit (Ugo Basile, Gemonio, Italy), consisting of an infrared radiant light source (100 W, 15 V bulb) focused onto a photocell utilizing an aluminum parabolic mirror. During the trials, the mice were gently hand-restrained using leather gloves. Radiant heat was focused 3–4 cm from the tip of the tail, and the latency (s) of the tail withdrawal to the thermal stimulus was recorded. The measurement was interrupted if the latency exceeded the cut off time (15 s). The baseline latency was calculated as mean of three readings recorded before testing at intervals of 15 min and the time course of latency determined at 15, 30, 45, 60, 90, and 120 min after treatment. Data were expressed as the area under the curve of the maximum percentage effect (%MPE) = (post-drug latency − baseline latency)/(cut-off time − baseline latency) × 100.

#### 3.8.5. Formalin Test

In the formalin test, the injection of a dilute solution of formalin (1%, 20 μL/paw) into the dorsal surface of the mouse hind paw evoked biphasic nociceptive behavioral responses, such as licking, biting the injected paw, or both, occurring from 0 to 10 min after formalin injection (the early phase) and a prolonged phase, occurring from 10 to 40 min (the late phase). Before the test, mice were individually placed in a Plexiglas observation cage (30 × 14 × 12 cm) for one hour, to acclimatize to the testing environment. The total time the animal spent licking or biting its paw during the early and late phase of formalin-induced nociception was recorded.

#### 3.8.6. Data Analysis and Statistics

Experimental in vivo data were expressed as mean ± s.e.m. Significant differences among the groups were evaluated with one-way ANOVA followed by Dunnett’s multiple comparisons test. GraphPad Prism 6.03 software was used for all the analyses. Statistical significance was set at *p* < 0.05. The data and statistical analysis comply with the recommendations on experimental design and analysis in pharmacology.

## 4. Conclusions

KORs play a crucial role in the pathogenesis of various disorders affecting the central nervous system, such as depression, anxiety, and pain. The stimulation of KOR leads to rather complex effects that reflect the structural complexity of this class of G protein coupled receptors. In silico experiments play an important role in the early stages of drug discovery, allowing one to reduce the time and costs associated with the identification of new molecules. In this experimental project, we used the computational technique of virtual screening to identify molecular structures that could show affinity for the KOR through significant interactions. In this phase, the virtual library, consisting of ~6 million molecules, was submitted to the HTVS rapid docking method. Among the 33 selected molecules, the H-D-Tyr-Val-NH_2_ dipeptide turned out to be of particular interest due to its structural requirements; therefore, it was considered for the next rational design step. In the drug design phase, we exploited knowledge about the KOR structure, designing tripeptides with higher docking score values than the JDTic crystallographic ligand and a more marked lipophilicity, with the aim of improving the pattern of hydrophobic interactions within the orthosteric receptor site. The most promising tripeptides were further analyzed through molecular dynamics simulations, which provided a more detailed picture of the evolution of tripeptide-KOR complexes. The entire in silico process furnished the necessary data to identify and estimate the most suitable compounds for synthesis and pharmacological tests.

The two tripeptides H-D-Tyr-Val-Val-O-(3-Br)-Bz and H-D-Tyr-Val-Trp-OBz contain Tyr as the first amino acid, which is essential for the interaction with the receptor, while leaving the stereochemistry of the initial dipeptide unchanged because it showed a greater affinity for KOR. The H-D-Tyr-Val-Val-O-(3-Br)-Bz structure was found to be the most stable within the receptor’s active site and recorded the highest docking score; these results are probably due to improved hydrophobic interactions involving important amino acid residues, such as Ile294, Val118, and Tyr312. The HD-Tyr-Val-Trp-OBz tripeptide does not show significant values in MD simulation analyses; however, it exhibits favorable stabilization in the receptor pocket due to additional hydrophobic interactions with Tyr139, Ile290, and Trp287, as well as a docking value score higher than JDTic. This shows structural similarities with the endogenous opioid tetrapeptide EM-2 (H-Tyr-Pro-Trp-Phe-NH_2_) selective on MOR. In vivo tests revealed their ability to induce an antinociceptive effect after i.c.v. and s.c. administrations in the tail flick and formalin tests, respectively. Among them, peptide **11** is active also in the second phase of the last test. This is somewhat in agreement with the ADME prediction, which indicates a possible inhibition of the drug-metabolizing cytochrome CYP3A4 and a low GIA absorption. However both of them show poor bioavailability, prompting us to further investigate their structural modifications with the aim of improving the pharmacokinetic properties and drug-likeness. Overall this experimental work allowed us to find interesting lead compounds for the next steps of structure optimization and pharmaceutical characterization.

## Figures and Tables

**Table 1:** Docking score values for the designed peptides based on the insertion of valine in third position (second approach).

Peptides Sequences	Docking Score
D-Tyr-L-Val-L-Val-OBz	−11.789
D-Tyr-D-Val-L-Val-OBz	−11.467
L-Tyr-L-Val-D-Val-OBz	−11.189
D-Tyr-D-Val-D-Val-OBz	−11.154
D-Tyr-L-Val-D-Val-OBz	−9.975
L-Tyr-D-Val-D-Val-OBz	−9.598
L-Tyr-L-Val-L-Val-OBz	−8.510
L-Tyr-D-Val-L-Val-OBz	−8.188

**Table 2:** Docking score values for the designed peptides based on the insertion of tryptophan in third position (second approach).

Peptides Sequences	Docking Score
D-Tyr-L-Val-L-Trp-OBz	−11.582
L-Tyr-D-Val-L-Trp-OBz	−11.075
D-Tyr-D-Val-L-Trp-OBz	−8.174
L-Tyr-L-Val-L-Val-OBz	−7.523

**Table 3:** Docking score values for the designed peptides based on the third approach.

Peptides Sequences	Docking Score
D-Tyr-L-Val-L-Val-O-(3-Br)-OBz	−11.288
D-Tyr-D-Val-L-Val-(3-Br)-OBz	−10.728
D-Tyr-L-Val-D-Val-O-(3-Br)-OBz	−9.849
L-Tyr-L-Val-D-Val-O-(3-Br)-OBz	−9.451
D-Tyr-D-Val-D-Val-O-(3-Br)-OBz	−9.150
L-Tyr-L-Val-L-Val-O-(3-Br)-OBz	−9.087
L-Tyr-D-Val-D-Val-O-(3-Br)-OBz	−8.792
L-Tyr-D-Val-L-Val-O-(3-Br)-OBz	−7.774

**Table 4:** in silico ADME study for peptides **6** and **11**.

Peptides	Lipophilicity		Drug-Likeness		Pharmacokinetics		
	TPSA (Å)	CLogP (o/w)	Bioavailability Score	Lipinski Filter	GIA	P-gp Substrate	CYP3A4 Inhibitor
6	132.37	2.59	0.55	Yes (1)	high	yes	no
11	148.16	2.46	0.55	Yes (1)	low	yes	yes

Abbreviations: CLogP (o/w), logarithm of compound partition coefficient between n-octanol and water; CYP3A4, cytochrome P450 3A4; GIA, gastrointestinal absorption; Lipinski filter (with number of violations in bracket); TPSA, topological polar surface area.

## Supplementary Materials

The following are available online, Figure S1: 1H-NMR of peptide **6**; Figure S2: LRMS of peptide **5**; Figure S3: 1H-NMR of peptide **11**; Figure S4: LRMS of peptide **10**; Figure S5: ADME prediction for peptide **6**; Figure S6: ADME prediction for peptide **11**.

## Sample Availability

Samples of the compounds **6** and **11** are available from the authors.
