# Supplementary material for: In Silico Identification of Tripeptides as Lead Compounds for the Design of KOR Ligands
Source: Molecules. 2021 Aug 6;26(16):4767. doi: 10.3390/molecules26164767 (PMC8399634; doi:10.3390/molecules26164767)
Supplement: Supplementary file 1 [file molecules-26-04767-s001.zip › molecules-1328977-supplementary.pdf]

In Silico Identification of Tripeptides as Lead Compounds for the Design of KOR Ligands

Azzurra Stefanucci [1], Valeria Iobbi [2], Alice Della Valle [1], Giuseppe Scioli [1], Stefano Pieretti [3], Paola Minosi [3], Sako Mirzaie [4], Ettore Novellino [5] and Adriano Mollica [1,*]

[1] Department of Pharmacy, University G. d'Annunzio Chieti, Via dei Vestini 31, 66100 Chieti, Italy; a.stefanucci@unich.it (A.S.); alice.dellavalle@unich.it (A.D.V.); giuseppe.scioli@unich.it (G.S.)
[2] Department of Pharmacy (DIFAR), University of Genova, 16128 Genova, Italy; valeria.iobbi@edu.unige.it
[3] Centro Nazionale Ricerca e Valutazione Preclinica e Clinica dei Farmaci, Istituto Superiore di Sanità, Viale Regina Elena 299, 00161 Rome, Italy; stefano.pieretti@iss.it (S.P.); paola.minosi@uniroma1.it (P.M.)
[4] Advanced Pharmaceutics and Drug Delivery Laboratory, Leslie L. Dan Faculty of Pharmacy, University of Toronto, 27 King's College Circle, Toronto, ON M5S 1A1, Canada; sako.biochem@gmail.com
[5] NGN Healthcare, Via Nazionale Torrette, 207, 83013 Mercogliano, Italy; ettore.novellino@unina.it
* Correspondence: a.mollica@unich.it

**Table of Contents**	**Pages**
Figure S1: 1H-NMR of peptide **6**	2-4
Figure S2: LRMS of peptide **5**	5
Figure S3: 1H-NMR of peptide **11**	6-8
Figure S4: LRMS of peptide **10**	9
Figure S5: ADME prediction for **6**	10
Figure S6: ADME prediction for **11**	11

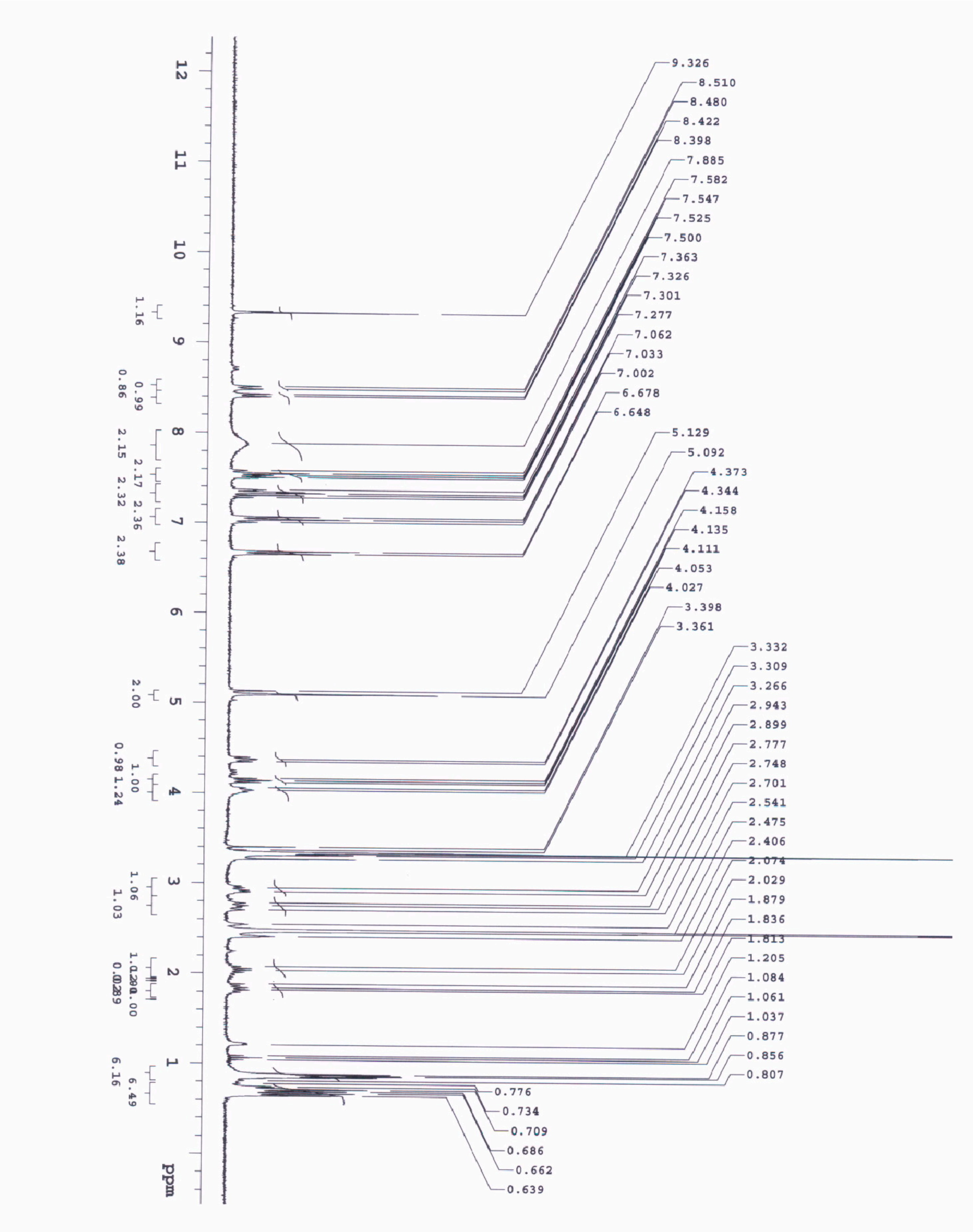
9.326
8.510
8.480
8.422
8.398
7.885
7.582
7.547
7.525
7.500
7.363
7.326
7.301
7.277
7.062
7.033
7.002
6.678
6.648
5.129
5.092
4.373
4.344
4.158
4.135
4.111
4.053
4.027
3.398
3.361
3.332
3.309
3.266
2.943
2.899
2.777
2.748
2.701
2.541
2.475
2.406
2.074
2.029
1.879
1.836
1.813
1.205
1.084
1.061
1.037
0.877
0.856
0.807
0.776
0.734
0.709
0.686
0.662
0.639
ppm

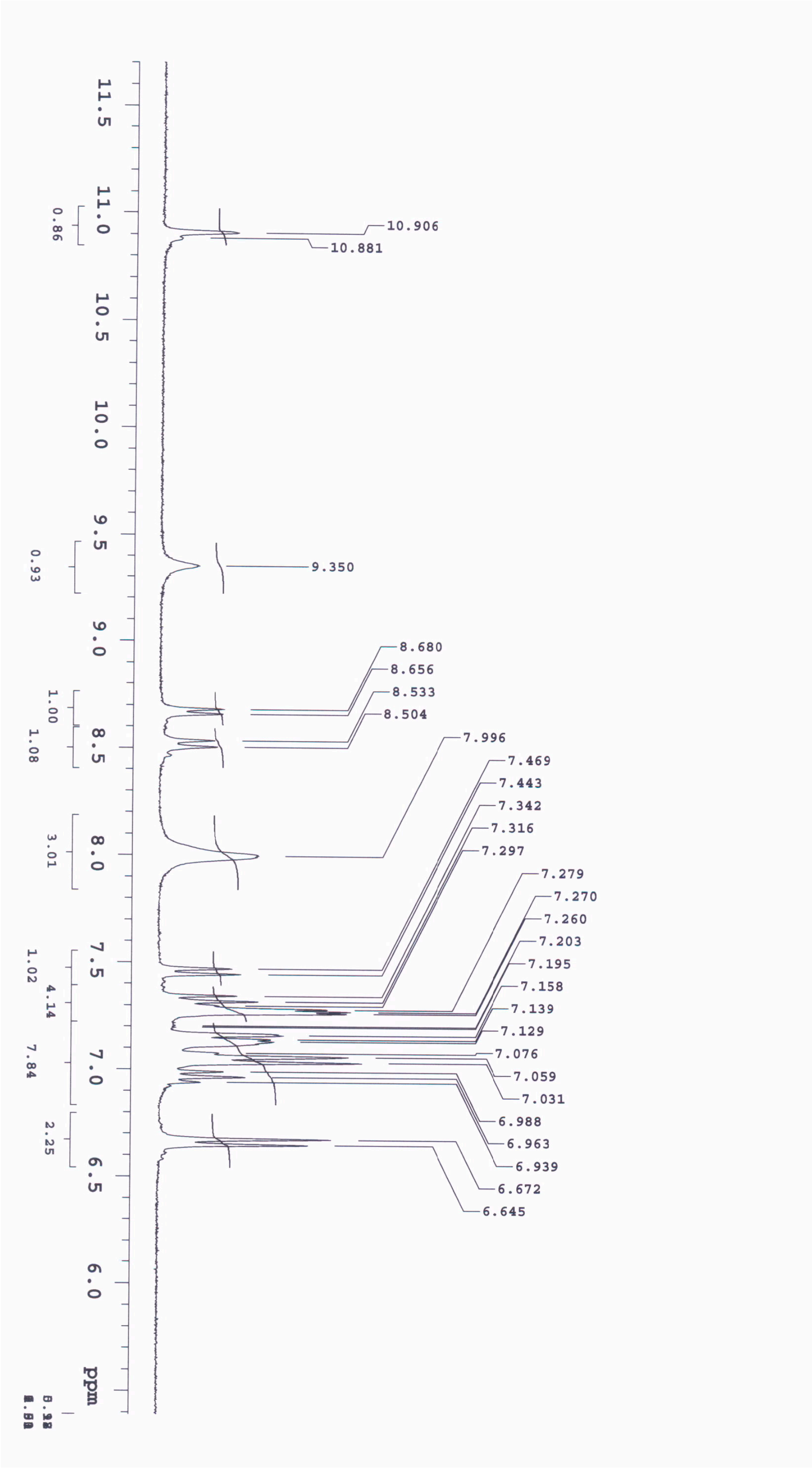
10.906
10.881
9.350
8.680
8.656
8.533
8.504
7.996
7.469
7.443
7.342
7.316
7.297
7.279
7.270
7.260
7.203
7.195
7.158
7.139
7.129
7.076
7.059
7.031
6.988
6.963
6.939
6.672
6.645
ppm

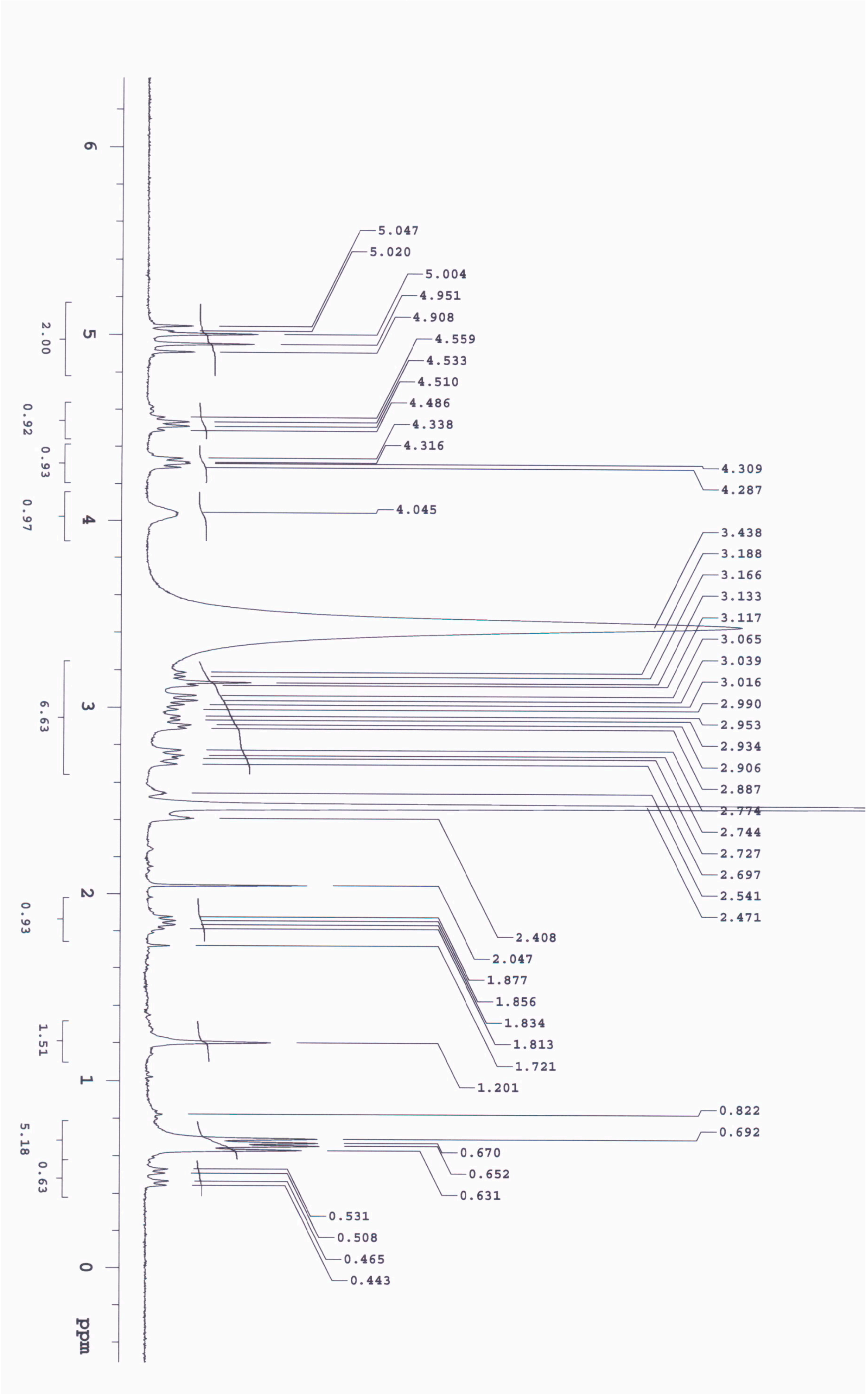

Figure S1. 1H-NMR of peptide **6** in DMSO-d_6.

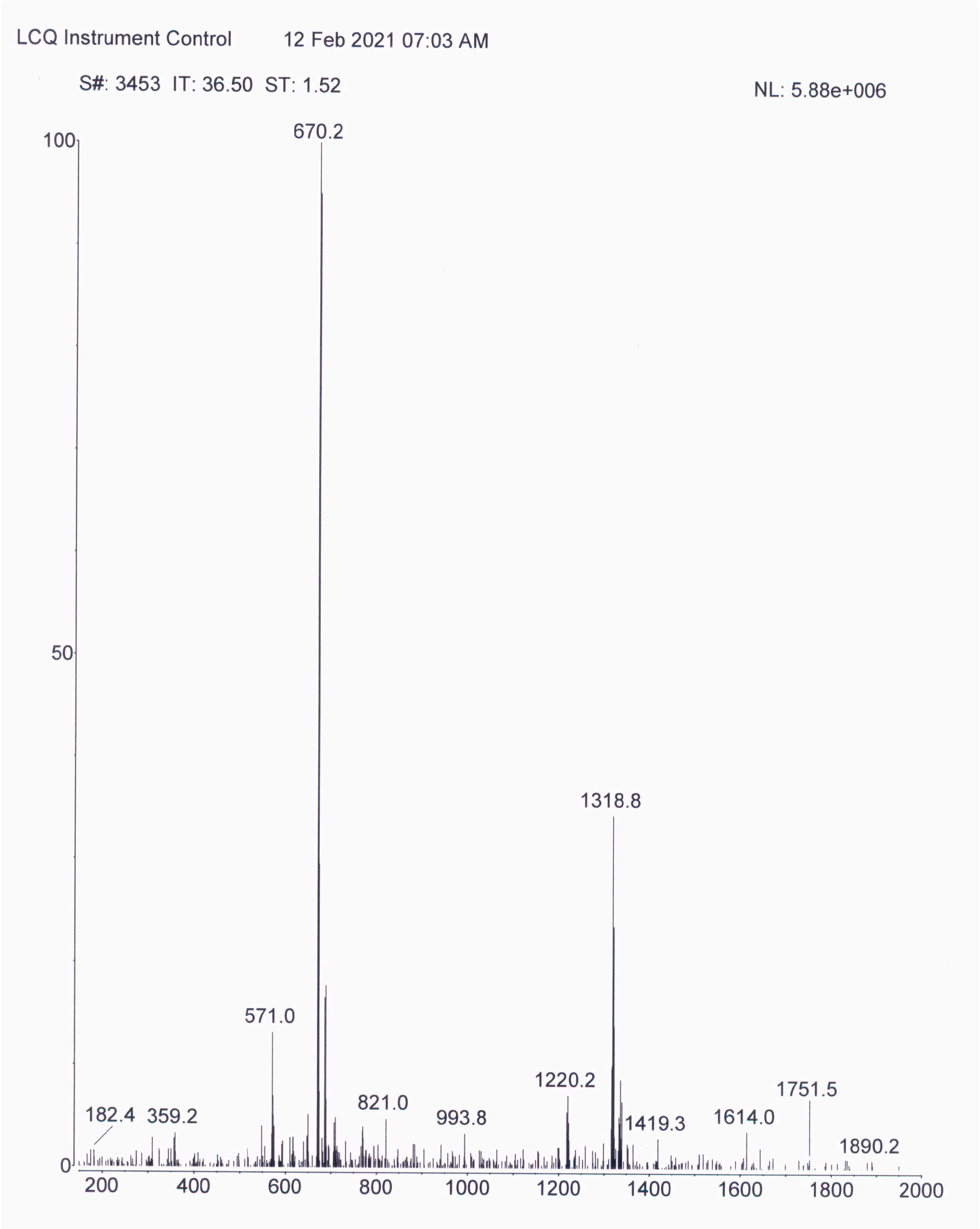

Figure S2. LRMS of peptide **5**.

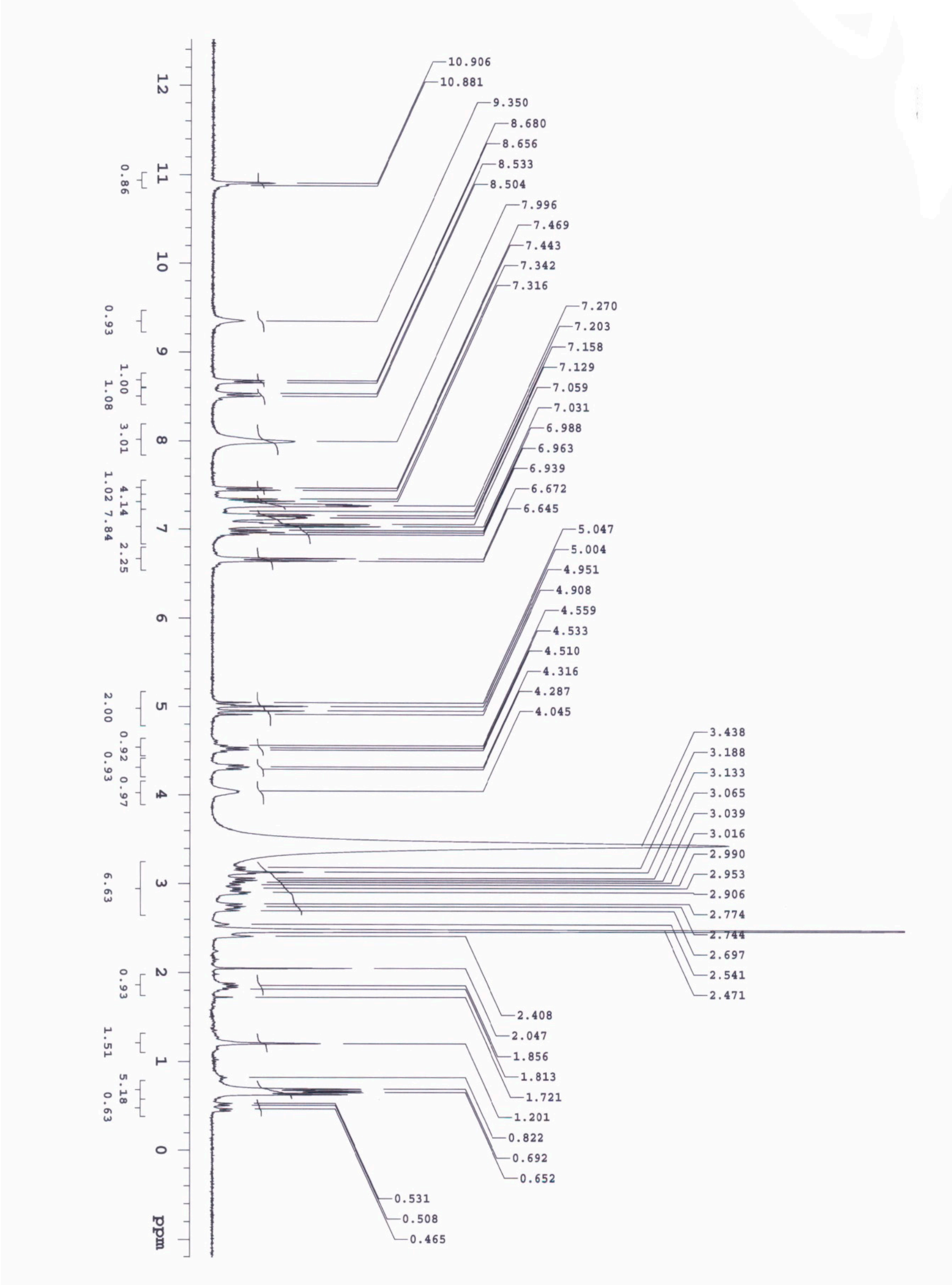
10.906
10.881
9.350
8.680
8.656
8.533
8.504
7.996
7.469
7.443
7.342
7.316
7.270
7.203
7.158
7.129
7.059
7.031
6.988
6.963
6.939
6.672
6.645
5.047
5.004
4.951
4.908
4.559
4.533
4.510
4.316
4.287
4.045
3.438
3.188
3.133
3.065
3.039
3.016
2.990
2.953
2.906
2.774
2.744
2.697
2.541
2.471
2.408
2.047
1.856
1.813
1.721
1.201
0.822
0.692
0.652
0.531
0.508
0.465
0.86
0.93
1.00
1.08
3.01
1.02
4.14
7.84
2.25
2.00
0.92
0.93
0.97
6.63
0.93
1.51
5.18
0.63
12
11
10
9
8
7
6
5
4
3
2
1
0
ppm

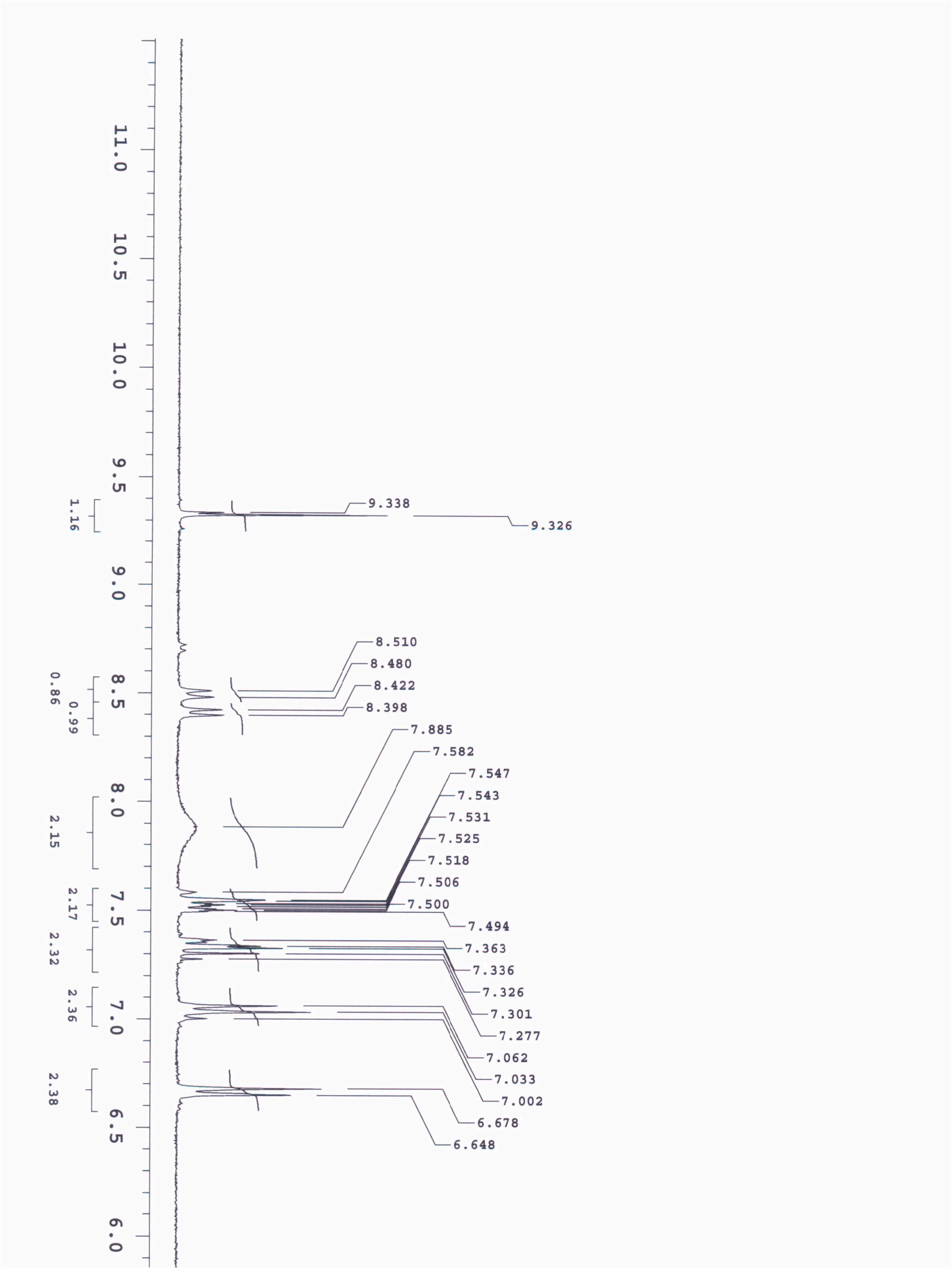
11.0
10.5
10.0
9.5
9.0
8.5
8.0
7.5
7.0
6.5
6.0
1.16
0.86
0.99
2.15
2.17
2.32
2.36
2.38
9.338
9.326
8.510
8.480
8.422
8.398
7.885
7.582
7.547
7.543
7.531
7.525
7.518
7.506
7.500
7.494
7.363
7.336
7.326
7.301
7.277
7.062
7.033
7.002
6.678
6.648

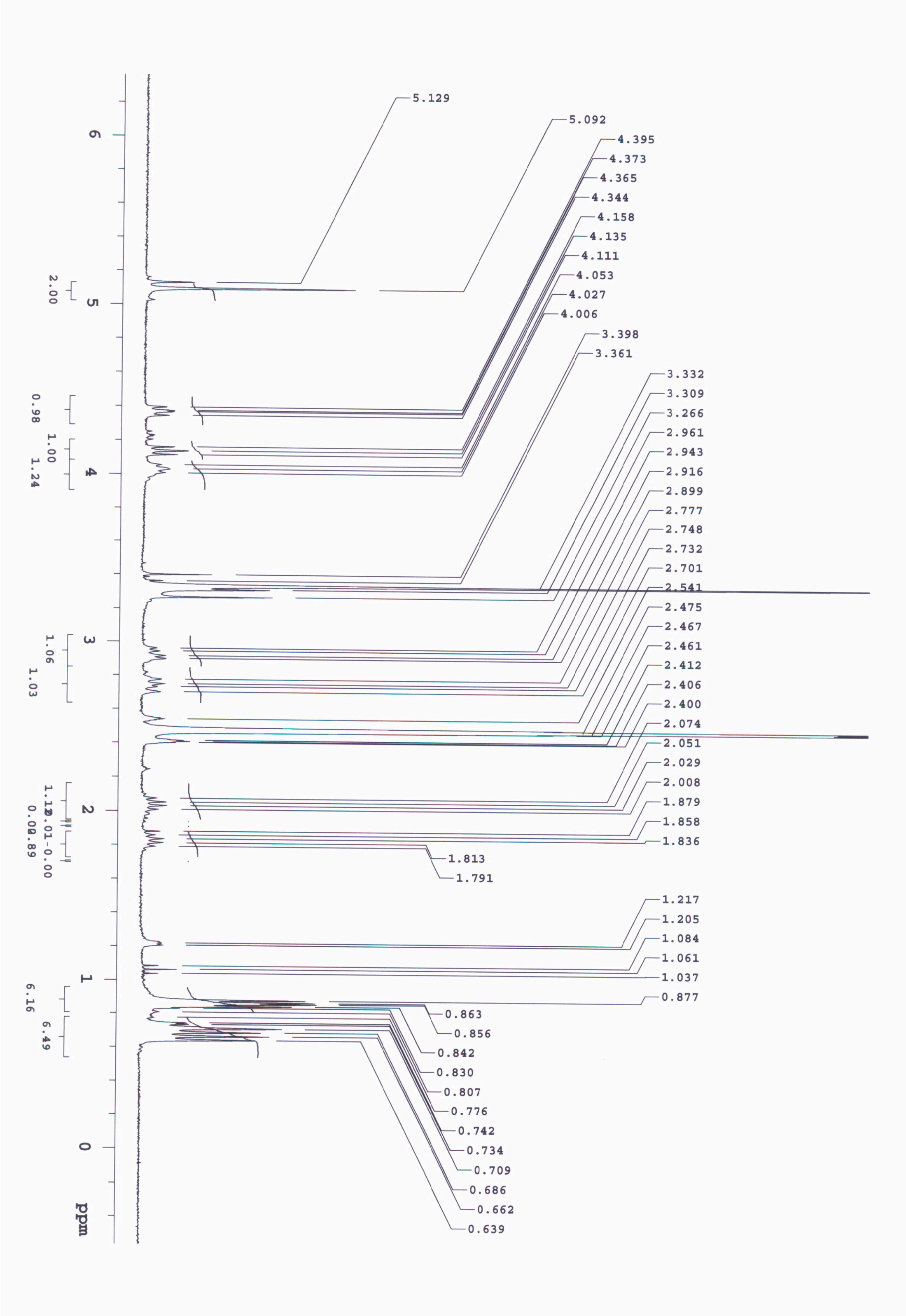

Figure S3. 1H-NMR of peptide **11** in DMSO-d_6.

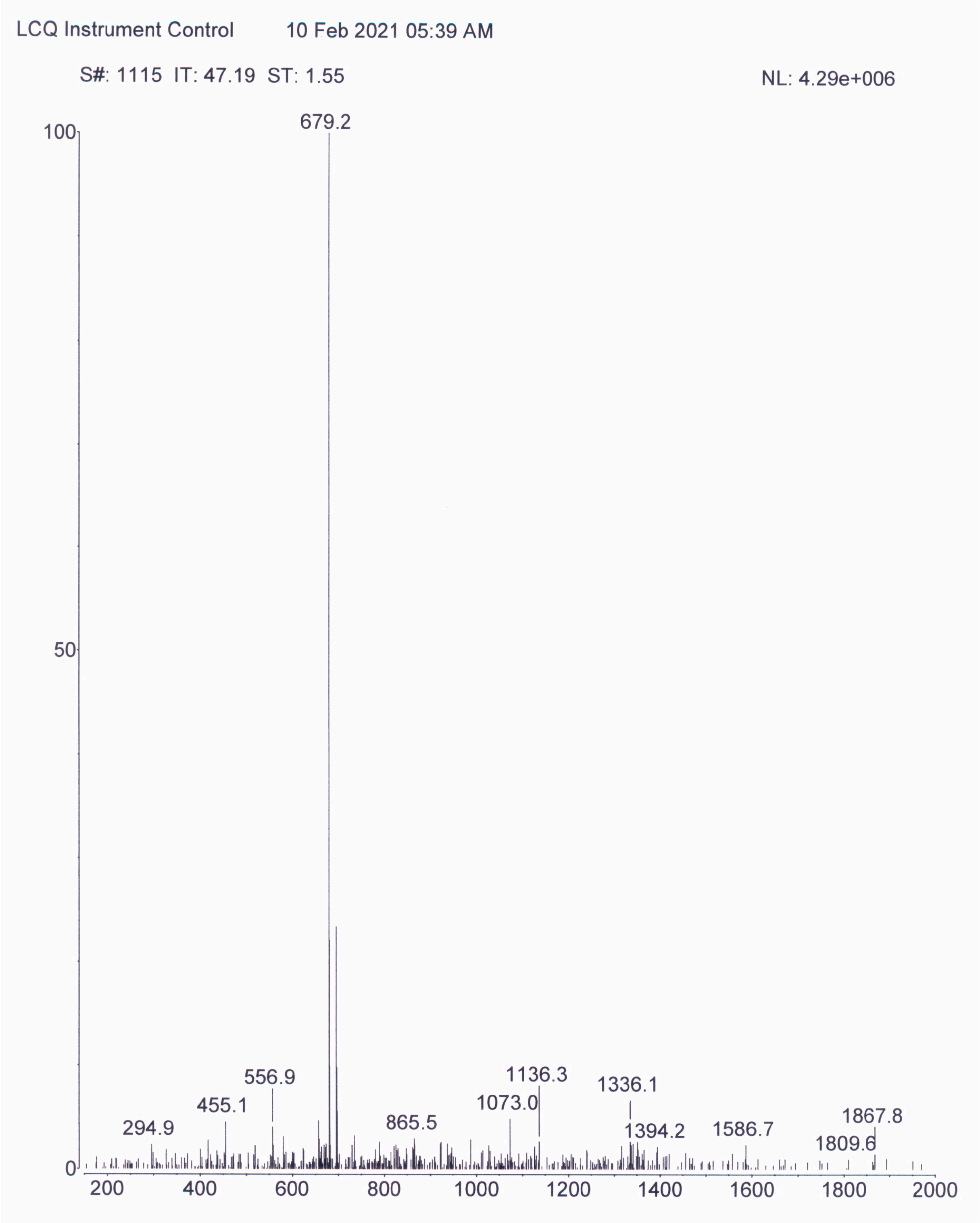

Figure S4. LRMS of peptide **10**.

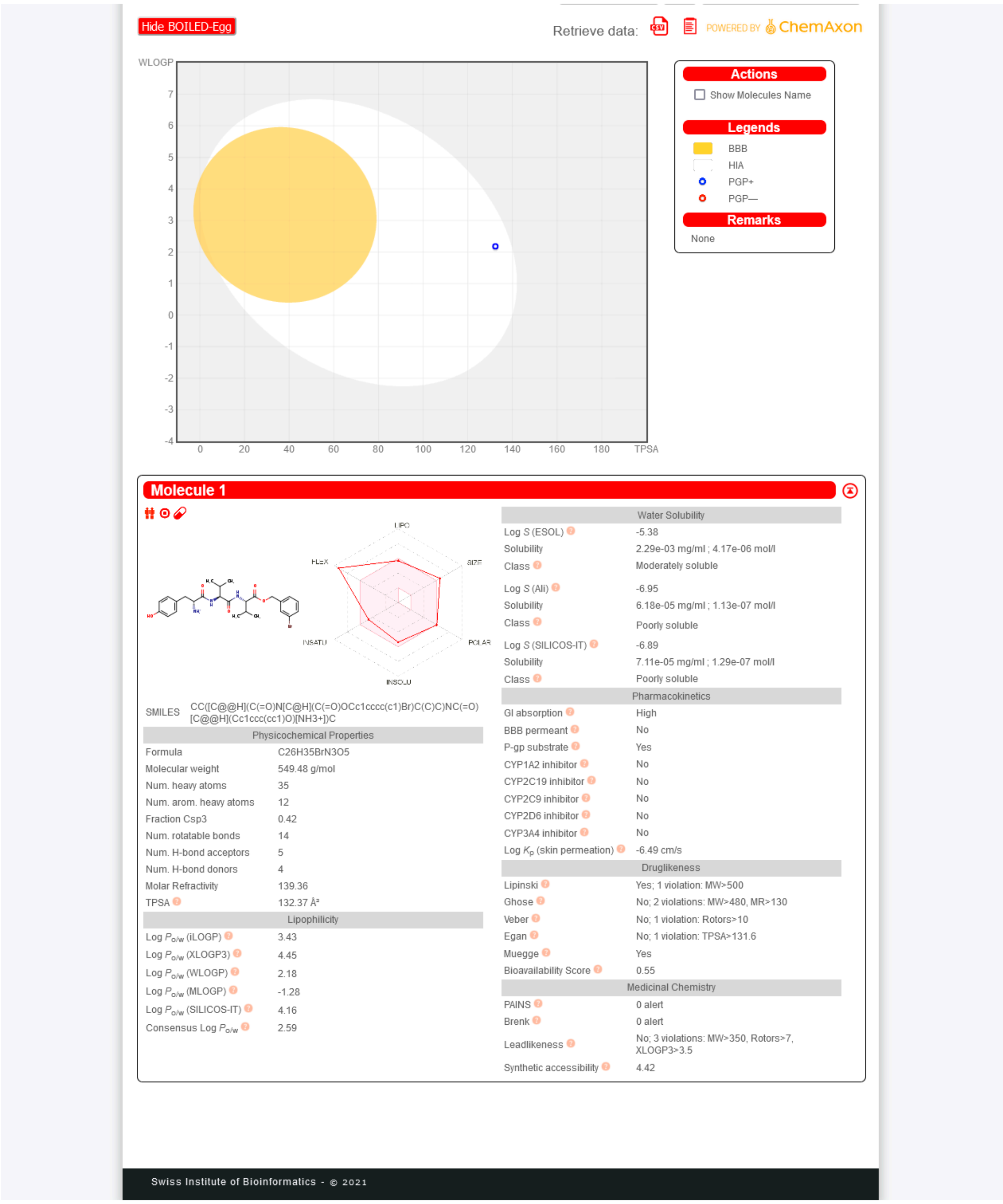

Figure S5. ADME prediction for **6**.

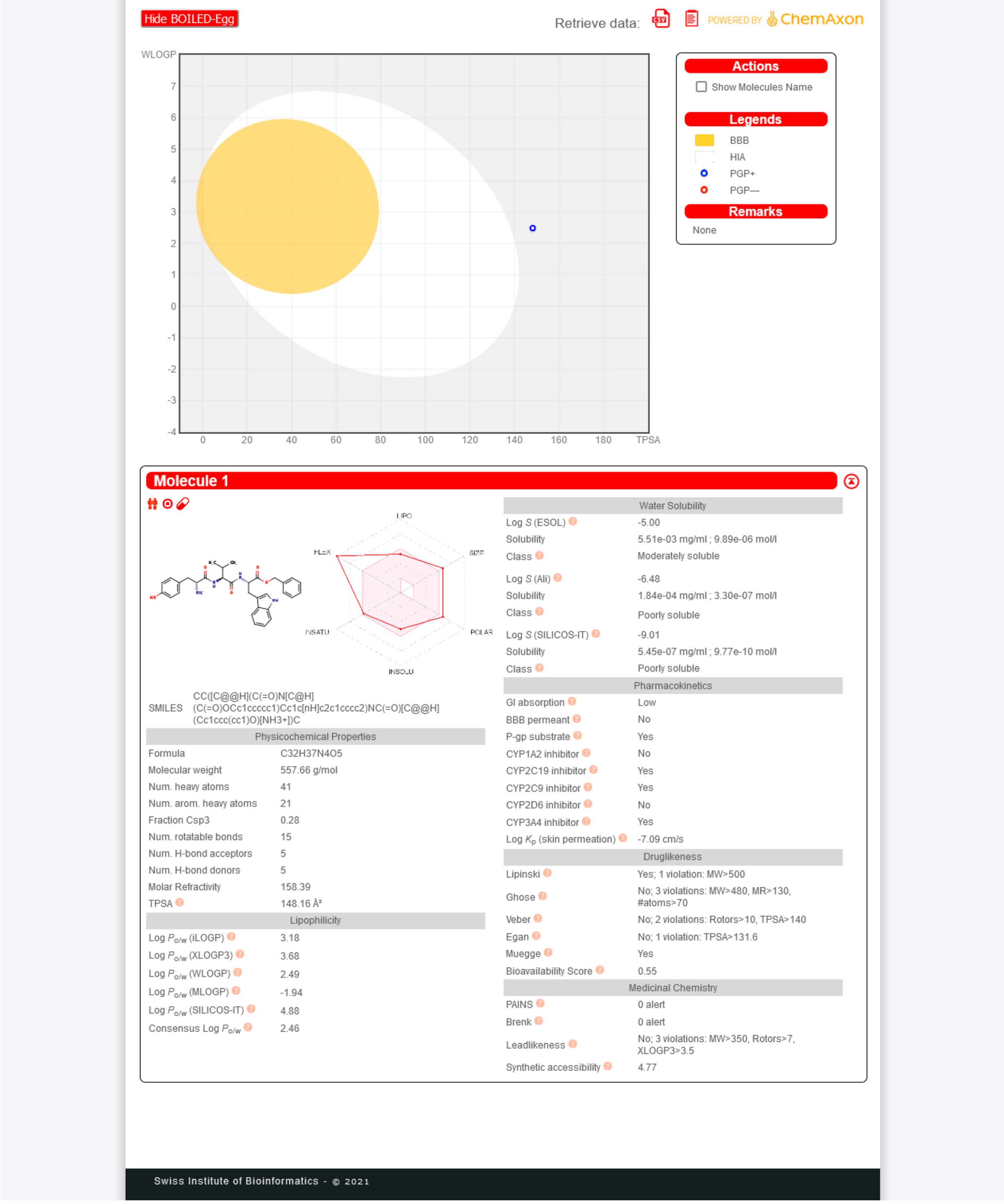

Figure S6. ADME prediction for **11**.
